# Dietary E‐Health Interventions for Adults With Severe Mental Illness: A Systematic Review

**DOI:** 10.1111/jhn.70112

**Published:** 2025-08-24

**Authors:** Kathy Holmes, Susan Heaney, Angela Smith, Janelle Skinner, Tracy Burrows

**Affiliations:** ^1^ Hunter New England Local Health District Maitland New South Wales Australia; ^2^ Department of Rural Health University of Newcastle Port Macquarie New South Wales Australia; ^3^ Food and Nutrition Research Program, Hunter Medical Research Institute New Lambton Heights New South Wales Australia; ^4^ Hunter New England Local Health District New Lambton Heights New South Wales Australia; ^5^ College of Health, Medicine and Wellbeing University of Newcastle Callaghan New South Wales Australia

**Keywords:** diet, e‐health, severe mental illness, systematic review

## Abstract

**Background:**

Delivery of dietary interventions using e‐health for severe mental illness populations show promise. Yet the details of what has been trialled and the effectiveness of such interventions in this population group has not been systematically reviewed.

**Objective:**

To systematically review and synthesise published research to determine the evidence of e‐health delivered nutritional interventions on diet, physical and mental health outcomes in adults living with severe mental illness.

**Design:**

Five electronic databases were searched to March 2025 to identify studies in adults living with severe mental illness, interventions including a dietary focus and delivered via e‐health, and reporting diet, mental health, or anthropometric outcome/s. The completeness of intervention reporting was evaluated using the TIDieR (Template for Intervention Description and Replication) framework.

**Results:**

Eleven studies (*n* = 1827 individuals, 56% female) were included, including randomised controlled trials (*n* = 6) and pre/post studies (*n* = 5). Most interventions were delivered by health professionals (*n* = 8 studies) and the duration was highly varied (6 weeks − 12 months). Anthropometrics were the most common outcome (*n* = 8 studies; with five reporting significant improvements) followed by physical activity (*n* = 6 studies; with two reporting significant improvements), mental health and cardiovascular outcomes (*n* = 5 studies each; with five and four of these studies, respectively, reporting significant improvements in outcomes). Diet was assessed in only three studies, one reporting significant changes. Ten studies incorporated smartphone technology including using phone calls (*n* = 5 studies), text messages (*n* = 3 studies), smartphone applications or multimodal delivery.

**Conclusion:**

This review identified opportunities to inform clinical service including more opportunity to harness other aspects of technology for the entire scope of the nutrition care process (e.g. better dietary assessment and use of features such as text message and assessing outcome measures).

## Summary

- While the delivery of dietary interventions using e‐health for severe mental illness (SMI) populations show promise, the evidence is limited to a small number of studies with anthropometrics, cardiovascular disease, physical activity, and mental health outcomes more commonly assessed than diet
- To further the field, future dietary interventions in adults living with SMI, should include assessment of dietary intakes to determine the resultant changes from dietary focused interventions and make use of all TIDieR framework criteria to improve reporting of interventions
- There is also opportunity to harness other aspects of e‐health technology for the entire scope of the nutrition care process such as better dietary assessment and use of features such as text messaging and assessing outcome measures

## 1 Introduction

Severe mental illness (SMI) refers to a subset of mental health disorders where there is a higher level of impact to function and quality of life (QoL) or higher loss in health [1]. This includes schizophrenia and bipolar disorder, which affects approximately 1 in 222 and 1 in 150 adults worldwide, respectively [2]. Individuals living with SMI not only experience higher rates of physical ill‐health than the general population, but also have a life expectancy that is 15–20 years shorter [3], and evidence suggests this gap may be widening [4]. Poor physical health for people with SMI are attributed to factors including food insecurity [5] in addition to antipsychotic medications and their impacts on metabolism [6]. With respect to dietary intakes of individuals with SMI, these have previously been reviewed highlighting poor diet and eating behaviours, characterised by comfort eating, high intakes of discretionary foods (i.e. foods high in fat, added sugar, added salt and/or kilojoules), sugar sweetened drinks, and low diet quality with low intakes of core foods groups such as fruit, vegetables and wholegrains [7]. These dietary intakes present modifiable risk factors and contribute to poor physical health and premature deaths in individuals with SMI that are often attributed to lifestyle diseases, such as cardiovascular disease (CVD) and diabetes [8]. A previous review (*n* = 58 studies) [7] demonstrated that there is a need for effective dietary interventions in individuals with SMI to avoid further widening of the mortality gap, with additional reviews demonstrating that interventions delivered by dietitians are more effective than when delivered by other health professionals [9, 10, 11]. The number of dietitians working in mental health care teams are increasing as their role is better defined and their skills in dietary assessment and behaviour change are recognised as having paramount importance in physical health [12, 13]. However, with the growing number of individuals with mental ill health, many dietetic services within public inpatient and outpatient facilities focus on those most acutely unwell leaving a large gap in nutrition support for many individuals with SMI in the community [1, 12].

E‐health (electronic health) interventions have grown rapidly in popularity and provide an opportunity for broader reach in the treatment of mental illness beyond clinical settings. This is largely due to reasons such as increased access in rural and regional areas, reduced anxiety around attending appointments, the ability to more easily re‐schedule appointments and send reminders through mediums such as text message [14], as well as cost reductions compared with face‐to‐face interventions [15]. Previously, there have been concerns that people with SMI may experience socioeconomic barriers such as unstable housing, low income, and unemployment which may limit their access to the internet and online interventions [16]. Encouragingly, however, smartphone and internet use is increasing among those with SMI [17], with smartphone ownership rates nearly as high as the general population [18, 19]. While these experiences of digital poverty may vary for people with SMI, particularly over time, disparities in levels of digital literacy also exist [20]. Digital skills training may be necessary to increase access to care through technology [21]. Previous research [22] demonstrates that digital approaches towards delivering health behaviour change in individuals with SMI were feasible and acceptable and can illicit change in behaviour and health outcomes [23].

The World Health Organisation defines digital health as the field of knowledge and practice associated with the development and use of digital technologies to improve health [24]. Meanwhile in 2020, Fatehi et al. conducted a systematic review of its definition and defined digital health as ‘the proper use of technology for improving the health and wellbeing of people at individual and population levels, as well as enhancing the care of patients through intelligent processing of clinical and genetic data’ [25]. On the other hand, the Australian Government Institute of Health and Welfare refers to it as an umbrella term for a range of technologies that can be used to treat patients and collect and share a person's health information. These can include the following: mobile health and applications (such as SMS reminders via mobile messaging, wellness apps, Medicare Online and COVID check‐in apps), electronic prescribing, electronic health records (including My Health Record), telehealth and telemedicine, wearable devices (such as fitness trackers and monitors), robotics and artificial intelligence [26]. Digital health, by any of these definitions, has become an integral part of the contemporary practice of medicine, with the COVID pandemic providing a catalyst for the rapid uptake of various forms of digital health since 2020 [27].

Improving the physical health of people living with mental illness, along with increasing equitable access to those living in rural and regional areas are identified priority areas. The Australian National Mental Health Commission ‘Vision 2030 Framework’ [28] identifies access to mental health services in all geographic locations and in all settings as one of the key focus areas. The framework highlights the case for change with flexible innovative practices guiding service provision into the future, including the need for increased digital and telehealth delivered mediums. A recent report also identifies significant gaps in knowledge of efficiency and effectiveness of online and telehealth services for rural communities, with a clear acknowledgement that health services for rural locations need to look at new models of service delivery to increase reach and improve health outcomes [29].

To date, there has been no synthesis of existing research of e‐health interventions that focus on dietary intakes in SMI. Additionally, the details of intervention characteristics related to what has been trialled remains unknown. The aim of this review is to synthesise the evidence of the effectiveness of e‐health delivered dietary interventions that focus on in improving dietary, mental health, anthropometric and physical health related (e.g., physical activity levels, CVD risk) outcomes among adults living with SMI. This includes describing the effectiveness of the intervention to improve outcomes as well as evaluating existing interventions against components of the TIDieR (Template for Intervention Description and Replication) [30] framework to identify research opportunities to inform clinical service.

## 2 Methods

This systematic review was registered with PROSPERO (CRD42023450091) https://www.crd.york.ac.uk/prospero/display_record.php?ID=CRD42023450091 and is reported in line with the Preferred Reporting Items for Systematic Reviews and Meta‐Analyses (PRISMA) 2020 guidelines [31].

### 2.1 Search Strategy

A robust search strategy was developed with the assistance of a clinical research librarian (AS), see Supporting Information S1: Table S1. The search strategy was developed based on the PICOS framework (Table 1). The search was developed in Medline before being translated to the other databases. A combination of controlled vocabulary and keyword search strings were included in each database search strategy. Five electronic databases; Cochrane (Wiley), PsycInfo (OVID), Cinahl (Ebsco), Medline (OVID), Embase (OVID), were searched systematically up to March 2025. References were exported from the databases into Covidence where duplicates were removed. Two researchers (TB, SH, KH, JS or CN) independently screened title and abstracts against the inclusion/exclusion criteria. Conflicts were resolved by a third researcher. Full texts for studies appearing to meet inclusion criteria were retrieved and underwent further screening by two researchers independently. Conflicts were resolved by a third researcher.

**Table 1:** PICOS framework and inclusion/exclusion criteria.

Study characteristics	Inclusion criteria	Exclusion criteria
Population	Adults ( ≥ 18 years) living with a diagnosis of severe mental illness (SMI)*	Individuals with a primary diagnosis of feeding and eating disorders, mild depression/anxiety without severe functional impairment, and neurodevelopmental disorders (such as autism, intellectual disability, ADHD) will be excluded.
	This includes: Individuals meeting DSM criteria for SMI (e.g. schizophrenia spectrum disorder, bipolar affective disorder, severe depression with/without psychotic features) Individuals with self‐reported SMI diagnoses, and studies where study authors define participants as having SMI (even if specific diagnoses are not provided) *Studies involving participants with multiple conditions/diagnoses will be included if more than 90% of participants have a clinical diagnosis of SMI or data limited to those with SMI are available	
Intervention	All dietary interventions, including lifestyle interventions with a dietary component AND Interventions delivered via e‐health, defined as either; sessions solely delivered via e‐health technology such as telephone, video, electronic messaging, mobile health apps, remote patient monitoring, or interventions where > 50% was delivered via e‐health	Interventions with no dietary component AND/OR Delivery of interventions where e‐health was not the focus/predominant type of delivery method (e.g., face‐to‐face sessions)
Comparison	All comparisons considered, this includes usual care or no control	NA
Outcomes	Primary outcomes: 1. Dietary outcomes (nutrients, food groups, eating behaviours, diet quality, dietary intake) OR 2. Mental health outcomes (change to clinical severity or presentation assessed via a validated tool) OR 3. Anthropometric outcomes (weight/BMI/waist circumference) OR 4. Other nutrition‐related health outcomes (e.g. cardiovascular disease risk) or lifestyle behaviour outcomes (e.g. quality of life, physical activity levels)	None of these outcomes
Study type	Intervention studies (e.g. randomised and non‐randomised controlled trials, pre‐post studies)	Qualitative studies, reviews, cross‐sectional studies, case studies, letters to the editor, books and thesis

### 2.2 Study Selection

To be included in this review articles needed to be in English, published anytime from database inception to the search date, and be an original peer‐reviewed journal article. These articles needed to report the dietary or mental health or anthropometric or lifestyle behaviour/nutrition‐related health outcomes of dietary interventions, or lifestyle interventions with a dietary component, and had to include adults diagnosed or classified by the study as having SMIs, which included but was not limited to schizophrenia spectrum disorders, bipolar affective disorders, depression with/without psychotic features. Additional inclusion criteria are listed in Table 1.

### 2.3 Risk of Bias Assessment

The appropriateness of the study designs, and the quality of how the studies were conducted, in the included articles were assessed to inform the level of risk related to potential biases (i.e. selection bias, performance bias/confounding, detection bias, attrition bias or reporting bias). Articles were quality checked for risk of bias by two independent reviewers (KH and CN) using a standardised, validated 10 item tool from the American Dietetic Association for human studies [32]. Studies were not excluded based on study quality. The quality criteria assessed 10 items relating to scientific soundness. The items assessed include: the research question, study groups and participants, outcome measures and statistical analysis. Each item was classified as present (‘Yes’), absent (‘No’), ‘Unclear’ or ‘Not Applicable’ for each included study. If most (six or more) of the answers to the quality questions were ‘No’, the study was designated with a negative (−) symbol. If the answers to quality criteria questions 2, 3, 6, and 7 did not indicate that the study was exceptionally strong, the study was designated with a neutral (ø) symbol. If most of the answers to the quality areas were ‘Yes’ (including criteria 2, 3, 6, 7 and at least one additional ‘Yes’), the study was designated with a positive (+) symbol.

### 2.4 Data Extraction and Analysis

Data were independently extracted by two researchers (KH or CN) into a purpose developed template. This was initially piloted for two studies to determine if it met the objectives of the review, or any changes were required. Extracted data included study and population characteristics, and intervention characteristics according to TIDieR [30] checklist criteria, and the relevant health behaviour outcomes. Outcomes were classified as anthropometrics [which included measures such as body weight, body mass index (BMI, kg/m^2^), waist circumference], mental health (which included symptoms scores, levels of severity, QoL), dietary (food, nutrients, energy, diet quality), physical activity (this included moderate/vigorous activities, sedentary behaviour), and cardiovascular health (which included cardiometabolic, blood lipids, blood pressure etc). Study outcomes are presented in a narrative summary.

### 2.5 Assessment of Intervention Reporting

The TIDieR framework [30] was used to evaluate the completeness of reporting of the interventions through the 12‐item checklist. This included a description of the intervention, the rationale, the completeness of the methods (materials, procedures, etc.), tailoring and modifications of the study, and the planned and actual performance of the study (especially regarding fidelity). Using TIDieR ensures that researchers provide comprehensive and transparent details about interventions, enabling others to replicate or build upon the study findings effectively. This promotes transparency, improves the quality of reporting, and facilitates the translation of research into practice. Evaluation of the intervention characteristics against TIDieR criteria was carried out by JS and checked by TB. Studies were allocated a colour code for each TIDieR criteria: green, indicated the study fully met the recommendations; amber, indicated the study partially met the recommendations; and red indicated the recommendation had not been met or the element was not included in the report [33]. Data are presented in narrative form.

## 3 Results

### 3.1 Description of Included Studies

**Figure 1:**
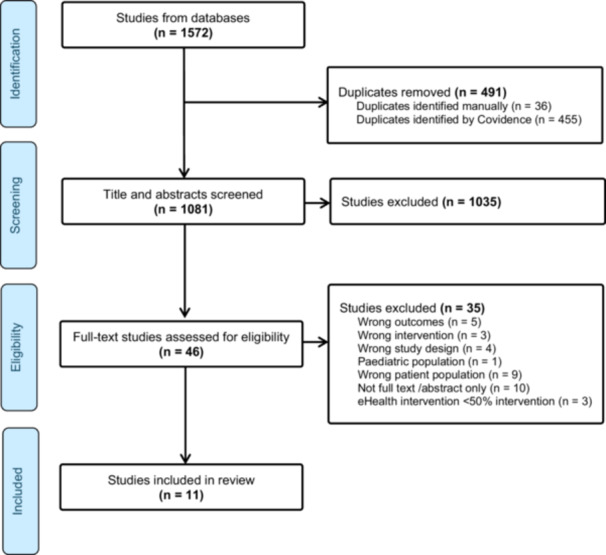
PRISMA flow diagram of article identification retrieval and inclusion for the systematic review.

A total of 1572 articles were retrieved from the search. Following removal of duplicates and screening against the inclusion criteria, a total of 11 studies [34, 35, 36, 37, 38, 39, 40, 41, 42, 43, 44] were included. Following full text screening the major reasons for exclusion were full text not available and wrong population group (Figure 1). The included studies were published between 2013 and 2023 with the majority being carried out in the United States of America (*n* = 5 [34, 35, 36, 37, 38]) and Australia (*n *= 3 [39, 40, 41]), (Table 2). Of the study designs, six were randomised controlled trials [34, 35, 39, 40, 42, 43], and five pre‐post studies [36, 37, 38, 41, 44]. The control groups across the RCTs included waitlist (*n* = 1 [34]), usual care (*n* = 1 [43]), basic education only (*n* = 1 [35]), face to face sessions (*n* = 2 [39, 40]), or same content without the supporting smart phone application applied (*n* = 1 [42]).

**Table 2:** Characteristics of included studies.

Author, year, country	Study objective	Study design | Study duration	Age range | Inclusion criteria	Recruitment setting	Sample size | Sex	Lifestyle outcome assessment
Abbott [34], 2020, USA	To determine the efficacy of a multimodal, online and community‐based lifestyle intervention for improving depressive symptoms and QoL	RCT | 6 weeks	20–64 y | MDD, BMI 18.4–34.9 kg/m^2^	Social media	*n* = 71 | 87% female	36‐Item Short Form Health Survey (SF‐36), Medical Symptoms Questionnaire (MSQ), Patient Health Questionnaire‐9 (PHQ‐9)
Aschbrenner [36], 2016a, USA	To evaluate the effectiveness of a peer‐group lifestyle intervention on weight and fitness outcomes	Pre‐post | 24 weeks	≥ 21 y | SMI, stable pharmacological treatment, BMI ≥ 30 kg/m^2^	Community MH centre	*n* = 32 | 56% female	Researcher measured body weight and BMI, 6‐minutewalk test (6MWT)
Aschbrenner [37], 2016b, USA	To evaluate the feasibility of a behavioural weight loss intervention enhanced with peer support and mHealth technology	Mixed methods (pre/post + qualitative) | 24 weeks	≥ 21 y | SMI, BMI ≥ 30 kg/m^2^	Community MH centre	*n* = 13 | 73% female	Researcher measured body weight and BMI, 6‐minutewalk test (6MWT)
Aschbrenner [35], 2022, USA	To evaluate the effectiveness of a group lifestyle intervention (PeerFIT) enhanced mHealth compared with one‐on‐one mHealth coaching on CVD risk reduction	RCT | 12 months	18–35 y | SMI, BMI ≥ 25 kg/m^2^	Community MH centres	*n* = 150 | 55% female	Researcher calculated BMI using participant self‐report body weight, blood test (lipids, HbA1c), BP, 6‐min walk test (6MWT), International Physical Activity Questionnaire (IPAQ)
Baker [41], 2014, Australia	To evaluate the feasibility of a telephone derived intervention, using motivational interviewing and cognitive behavioural strategies, to improve diet and physical activity	Pre/post | 12 weeks	≥ 18 y | SMI, < 7 serves F&V/day, > 2 h sedentary/day	Australian Schizophrenia Research Bank	*n* = 20 | 47% female	Australian Recommended Food Score (ARFS), Beck Depression Inventory, World Health Organisation EUROHIS scale, Global Assessment of Functioning (GAF), screentime (min/day), International Physical Activity Questionnaire (IPAQ), Timeline Follow Back alcohol use subscale, Opiate Treatment Index cannabis subscale
Baker [40], 2015, Australia	To examine the efficacy of nicotine replacement therapy plus either a predominantly telephone delivered or face‐to‐face intervention for smoking cessation and CVD risk reduction	RCT | 12 months	≥ 18 y | SMI, regular antipsychotic medications, smoker > 15/day	Health service referral, media campaigns, & other research programmes	*n *= 235 | 41% female	Australian Recommended Food Score (ARFS), Brief Psychiatric Rating Scale (BPRS‐24), Beck Depression Inventory (BDI‐II), Global Assessment of Functioning (GAF), Impact of Weight on Quality of Life (IWOQOL‐Lite), SF‐12 Mental Component Scale (MCS), SF‐12 Physical Component Scale (PCS), International Physical Activity Questionnaire (IPAQ), ASSIGN score (CVD risk; calculated from age, gender, total cholesterol, high‐density lipoprotein, systolic blood pressure, diabetes, family history of heart disease, cigarettes per day)
Baker [39], 2018, Australia	To examine the efficacy of nicotine replacement therapy plus either a predominantly telephone delivered or face‐to‐face intervention for smoking cessation and CVD risk reduction	RCT | 36 months	≥ 18 y | SMI, regular antipsychotic medications, smoker > 15/day	Health service referral, media campaigns, & other research programmes	*n* = 235 | 41% female	24 h eating habits recall with overall diet score/unhealthy eating index created (index ranged from 0‐12, with higher scores indicating more unhealthy eating habits), Brief Psychiatric Rating Scale (BPRS‐24), Beck Depression Inventory (BDI‐II), Global Assessment of Functioning (GAF), Impact of Weight on Quality of Life (IWOQOL‐Lite), SF‐12 Mental Component Scale (MCS), SF‐12 Physical Component Scale (PCS), International Physical Activity Questionnaire (IPAQ), ASSIGN score (CVD risk; calculated from age, gender, total cholesterol, high‐density lipoprotein, systolic blood pressure, diabetes, family history of heart disease, cigarettes per day), researcher measured waist circumference, body weight and BMI
Lee [42], 2020, South Korea	To evaluate the effect of a therapeutic lifestyle change mentoring program using a smartphone application in outpatients, to structured mentoring in inpatients, on cardio‐metabolic factors	Pre/post + nonequivalent control | 28 weeks	≥ 20 y | schizophrenia, stable medication, stable body weight in past month	Community rehabilitation centres & psychiatric ward	*n* = 31 | 26% female	Researcher measured waist circumference and BMI, blood test (lipids)
Looijmans [43], 2019, Netherlands	To evaluate the effectiveness of a multimodal lifestyle approach, including a web‐based tool, to improve cardiometabolic health versus care‐as‐usual	RCT | 12 months	NR | SMI + one of the following: waist circumference > 88/102 cm (female/male), BMI > 25 kg/m^2^, fasting glucose > 5.6 mmol/L, HbA1c > 5.7%	Community MH treatment organisation	*n* = 244 | 51% female	Researcher measured waist circumference and BMI, metabolic syndrome z‐score (derived from blood test ‐ lipids, glucose, HbA1c; BP; &/or lipid‐lowering & antihypertensive medication use)
Nicol [38], 2022, USA	To test the feasibility and usability of an interactive obesity treatment approach that incorporates text messages to supplement monthly in‐person health coaching	Pre/post | 12 weeks	16–75 y | SMI, BMI > 28 kg/m^2^	Community clinic	*n* = 26 | 60% female	Researcher measured body weight, Clinical Global Impression–Severity (CGI‐S), Loss of Control Over Eating Scale (LOCES)
Temmingh [44], 2013, South Africa	To evaluate a telephonically delivered lifestyle coaching intervention aimed at weight reduction and wellness improvement	Pre/post | 12 months	> 18 y | SMI, stable in community	Referrals to wellness program	*n* = 761 | 72% female	Self‐reported body weight and waist circumference, researcher calculated BMI, general health using self‐rated scale

Abbreviations: BMI, body mass index; BP, blood pressure; CVD, cardiovascular disease; F&V, fruit & vegetables; HbA1c, glycated haemoglobin; MDD, major depressive disorder; MH, mental health; mHealth, mobile health; NR, not reported; QoL, quality of life; RCT, randomised controlled trial; SMI, severe mental illness; txt msg, text message.

### 3.2 Description of Participants

Across the included studies there were 1827 participants (mean age 41.2 ± 12.6 years; 56% female), with sample sizes ranging from 13 to 761 (Table 2). The majority of studies were inclusive of adults regardless of age, one study was specifically in young adults 18–35 years [35] and one study [38] included participants where the minimum age was 16 years old, however was included as the mean age fitted the inclusion criteria. All participants across studies were free living in the community. Nine studies [35, 36, 37, 38, 39, 40, 42, 43, 44] reported participants were receiving varying levels of mental health care support, outside of the study, ranging from low to high intensive treatment. The majority of studies included a broad range of SMI as inclusion criteria, four studies included a range of SMIs [35, 36, 37, 43], one study included Axis 1 psychiatric disorders requiring treatment with psychotropic medication (95% of participants had a diagnosis of bipolar or unipolar mood disorder or schizophrenia spectrum disorder) [44], five studies specifically included bipolar disorder [36, 37, 38, 39, 40], six included schizophrenia spectrum disorders [36, 37, 38, 39, 40, 42], and four included major depressive disorder [34, 36, 37, 38]. One study [38] included two participants with attention deficit‐hyperactivity disorder or autism spectrum disorder, however was included as the majority (92%) of the sample had a diagnosis of either schizophrenia, bipolar disorder or major depressive disorder. Six studies used BMI as an inclusion criterion, five of these studies [35, 36, 37, 38, 43] included participants with overweight and/or obesity as defined by BMI criteria (cut‐off points specified by studies were ≥ 25 kg/m^2^ [35], > 25 kg/m^2^ [43], > 28 kg/m^2^ [38], and ≥ 30 kg/m^2^ [36, 37]). Of these, two studies included participants with obesity only [36, 37], and one study [34] included a BMI range of 18.4–34.9 kg/m^2^.

### 3.3 Risk of Bias of Included Studies

Eight studies scored a positive (+) quality rating, while three studies scored a neutral (ø) quality rating. No study scored a negative (−) quality rating. All studies clearly stated the research question, and clearly described the focus of the intervention and comparators, and withdrawals (i.e., dropouts, lost to follow up, attrition rate), and reported appropriate conclusions that considered biases and limitations. The quality criteria that were missing or not described well for the studies included study participants free from bias (*n* = 1), study groups comparable (*n* = 1), blinding (*n* = 1) and outcomes not well reported (*n* = 1), (see Supporting Information S1: Table S2 for quality rating scale).

### 3.4 Summary of Interventions

The duration of the intervention was 12 months in three studies [35, 43, 44], 9 months in two studies [39, 40], 24 weeks in two studies [36, 37], 12 weeks in three studies [38, 41, 42], and 6 weeks self‐directed in one study [34] (Table 3). The number of follow‐up timepoints for outcome assessment ranged from one to six, and time to final follow‐up ranged from 9 weeks after baseline to 36 months after baseline. Across the included studies attrition rates ranged from 4% to 43% (median 26%) with higher attrition rates (29%–43%) reported in those with study follow‐ups of 12 months or longer (*n* = 5 studies [35, 39, 40, 43, 44]).

**Table 3:** Summary of intervention characteristics as per TIDieR criteria^a^ checklist [24].

Author, year	BRIEF NAME	WHAT Intervention type | Intervention focus	WHAT eHealth technology components	WHAT Control group	WHO PROVIDED intervention | WHERE intervention occurred	HOW intervention was delivered	WHEN and HOW MUCH Intervention duration | Intervention intensity/dose	TAILORING personalisation of intervention	HOW WELL Intervention fidelity (planned and actual) ‐ Training of facilitators | Intervention adherence
Abbott, 2020 [34]	Multimodal, online lifestyle intervention	Self‐directed, standardised lifestyle education | Diet ( ↑ core and ↓ processed foods), physical activity, sleep, tobacco cessation, social connection, MH	Online program + online community group	Waitlist control	Self‐directed learning | Online	Online education, communication with other participants via private online group	6 weeks | Self‐paced	No	NR | NR
Aschbrenner, 2016a [36]	Peer‐group lifestyle intervention enhanced with mHealth technology	Group weight management and exercise sessions | Diet (healthy eating for 5% weight reduction), physical activity ( ↑ gradually to 150 min/week), social support, self‐monitoring	Private Facebook group + Fitbit activity tracker + txt msg	N/A	Lifestyle coaches (weight management sessions), certified fitness trainer (exercise sessions) | Community MH centre and online	Face‐to‐face: experiential and collaborative weight management education sessions, group exercise sessions. Online: private Facebook peer network. Self‐monitoring with Fitbit. Txt msg: reminders and motivational msg.	24 weeks | 1 ×60 min weight loss session/week, 2 x optional group exercise/week, Fitbit and Facebook self‐directed, 2‐3 x txt msg/week	Participants worked together to develop plans to apply new information about healthy eating and exercise in their daily lives	Lifestyle coaches attended initial 2‐day training, weekly supervision sessions, and twice‐monthly follow‐up training on eHealth technology components | Mean weight management sessions attended = 16 out of 24 (median = 18). Mean optional exercise group sessions attended = 11.2 ± 12.8 = 28% attendance rate. All participants used Fitbit and 76% used the private Facebook group.
Aschbrenner, 2016b [37]	Behavioural weight loss intervention enhanced with peer support and mHealth technology	Group weight management and exercise sessions with MH support | Diet (monitoring dietary intake, restricting calories, ↓ fat intake), physical activity ( ↑ gradually to 150 min/week)	Private Facebook group + Fitbit activity tracker + txt msg	N/A	MH counselling students with MH nurse support, dietitian, wellness peer, certified fitness trainer | Community MH centre and online	In person: weight management education sessions and group exercise sessions. MHealth: Fitbit to track steps and distance, rewards milestones. Txt msg: attendance and physical activity reminders. Private Facebook group: sharing successes and healthy eating tips with each other	24 weeks | 1 ×90 min weight management group session, twice weekly exercise group (optional), Fitbit, Facebook, txt msg (regular – not further defined) to increase motivation and self‐ monitoring	Participant were given a personal calorie goal and a dietary fat intake goal to achieve a 1–2 pound per week weight loss	MH counselling students attended initial 2‐day training, and weekly supervision sessions. Fidelity to study protocol monitored through observation at each session by principal investigator. | Treatment attendance, researcher tracked ‐ overall poor attendance rate = 56% (79% during weeks 1‐12% and 33% during weeks 13‐24). Mean core weight management sessions attended = 13.5 ± 4.6 (out of 24). Mean optional exercise group sessions attended = 10 ± 10.6 (out of 45).
Aschbrenner, 2022 [35]	PeerFIT (group lifestyle intervention with mHealth)	Group weight management and exercise sessions | Diet ( ↓ calorie intake, SSB, processed foods, ↑ F&V, lean meats), ↑ physical activity	Private Facebook group + Fitbit activity tracker + txt msg	Education (weight loss and physical activity) + activity tracking. Phone calls (5 ×30 min calls/month), txt msg (3‐5/week), and activity trackers.	MH or fitness professional with Bachelor degree in related discipline | Community MH centre and online	Face‐to‐face: group lifestyle and exercise sessions. Online: private Facebook group, activity trackers, txt msg.	12 months | Intensive phase (6 months): 1 ×60 min group lifestyle session/week + 1 ×60 min group exercise session/week, Facebook group, activity trackers, 3‐5 txt msg/wk. Lower intensity phase (7‐6 months): weekly lifestyle sessions ceased	Intervention derived from an existing program and tailored to suit young adults. Participants set weight‐loss and physical activity goals.	Peer‐ FIT coaches attended initial training, and supervised during weekly meetings by study team and during once‐monthly meetings with certified fitness trainer. Study team monitored fidelity via checklist. | NR
Baker, 2014 [41]	*Better Health Choices* telephone‐intervention (pilot outcomes)	One‐on‐one behaviour change telephone sessions | Diet ( ↑ F&V consumption), ↓ sedentary behaviour and/or ↓ alcohol and/or ↓ smoking use; MH monitoring (mood, self‐harm thoughts, withdrawal, medication side‐effects)	Telephone calls	N/A	Clinical psychologists and psychology interns | Telephone	Telephone calls with psychologist providing feedback including baseline F&V consumption, and motivational interviewing for described behaviour changes	4 or 8 weeks | 8 x manual‐guided telephone sessions (1 or 2 sessions/week)	Participants set their own goals with targets guided by national guidelines and current CVD risk behaviour research	Intervention sessions audio‐recorded. Therapist competence rated using 11‐item validated Behaviour Change Counselling Index, score range 0–4. Mean score (n = 76 sessions) was 2.41 ± 0.26. Behaviour change skills used between “To some extent” and “A good deal” of the time. No significant difference between therapists (F_(5, 70)_ = 0.52, *p* = 0.758). | Treatment attendance (method NR) = 95%
Baker, 2015 [40]	*Better Health Choices* telephone‐intervention (outcomes to 12 months)	One‐on‐one behaviour change telephone sessions | Education on nicotine replacement therapy, nicotine and symptoms, medication and cognition, diet ( ↑ fibre, ↓ dietary fat, 7 serves F&V/day, hydration), ↑ physical activity	Telephone calls	Content via 16 ×1‐h face‐to‐face sessions, with same initial session and nicotine replacement therapy delivery as telephone intervention group	Psychologists | Face‐to‐face across 3 sites in Australia and telephone	Telephone calls with psychologist to monitor the patients use of nicotine replacement therapy, smoking use, withdrawals, side effects, symptoms of psychosis and mood	9 months | Initial face‐to‐face education session on smoking and CVD risk factors, 14 ×10 min phone calls, 30 min face‐to‐face nicotine replacement therapy delivery in weeks 4 and 8	Participants set healthy eating goals and goals around risk behaviours considered most problematic	Therapists received weekly clinical supervision, random subsample of face‐to‐face and telephone sessions audio recorded and rated on a six‐point scale (0‐5) using Cognitive Therapy Scale (mean score 3.71 ± 0.78) and therapy adherence rates ranged from 89.4 – 93.3%). | Treatment attendance (measure NR) – intervention participants had high attendance (i.e. attended 9 – 17 sessions) compared with control group (67% vs 48%).
Baker, 2018 [39]	*Better Health Choices* telephone‐intervention (outcomes to 36 months)	One‐on‐one behaviour change telephone sessions | Education on nicotine replacement therapy, nicotine and symptoms, medication and cognition, diet ( ↑ fibre, ↓ dietary fat, 7 serves F&V/day, hydration), ↑ physical activity	Telephone calls	Content via 16 ×1‐h face‐to‐face sessions (9 months) + 7 weekly sessions + 3 x fortnightly sessions + 6‐monthly sessions to 36 months	Psychologists | Face‐to‐face across 3 sites in Australia and telephone	Telephone calls with psychologist to monitor nicotine replacement therapy, withdrawals, side effects, and MH	9 months | Initial 90 min face‐to‐face session, 10 min telephone calls (weekly sessions for 8 wks, 3 x fortnightly sessions, monthly sessions for 6 months), face‐to‐face sessions in weeks 4 and 8	Participants set goals with regular monitoring. Barriers identified and problem solved.	Fidelity methods and results as reported by Baker et al. 2015 [27]
Lee, 2020 [42]	Therapeutic lifestyle change (TLC) mentoring program	Self‐directed, standardised lifestyle education with one‐to‐one mentoring support | Diet (suggested effective diet for obesity control), physical activity (customised walking plan ≥ 30 min/day), encouraged healthy habits alcohol intake, smoking and sleep	Smartphone app	Content per intervention, but delivered by health mentor	Adult health mentors | Community rehabilitation centre	App provides daily information to follow diet, creates walking plan and reminds participants to follow plan	12 weeks | Program following app schedule	No	NR | NR
Looijmans, 2019 [43]	LION (Lifestyle Interventions for Outpatients with serious mental illness in the Netherlands)	One‐on‐one in‐person sessions | Personalised plan for diet, physical activity, medication use, hygiene, stressors, substance use, sexual behaviour	Web tool – Traffic Light Method for Somatic Screening and Lifestyle	Care‐as‐usual	MH nurses | Screening and progress visits in participants living environment	Web tool: creates personal risk profile for each behaviour and constructs plan. Follow‐up visits: Evaluate progress using reports and adjust goals.	12 months | Initial screening and goal setting, fortnightly 15 min nurse visits to evaluate progress towards goals for first 6 months. After 6 months screen again and revisit goals, fortnightly 15 min nurse visits to track progress of new goals for final 6 months.	Participants set SMART goals for lifestyle behaviours. Progress tracking and adjustment of goals.	Nurses received initial 1‐day training and evaluation session after 3‐months to discuss barriers | Participants who completed at least one lifestyle behaviour screening and constructed a lifestyle plan with lifestyle goals were considered low users when no follow‐up reports were completed; a medium user when between 1 and 9 follow‐up reports were completed; and a high user when ≥ 10 follow‐up reports were completed. Low user n = 13 (12%), medium user n = 60 (56%) and high user n = 35 (32%).
Nicol, 2022 [38]	iOTA (Interactive obesity treatment approach)	One‐on‐one in‐person sessions or group sessions + brief one‐on‐one in‐person sessions | Diet (energy balance and meal planning), ↑ physical activity	Telephone calls + txt msg	N/A	Health coach | Outpatient or community setting with face‐to‐face and telephone check‐ins and txt msg	In‐person education on health behaviours, meal‐planning and nutrition, physical activity, and long‐term behaviour change. In‐person goal tracking, overcoming barriers and setting new goals. Telephone check‐ins for questions and motivation. Txt message health tips directly related to selected goals.	12 weeks | Monthly in‐person sessions with health coaches. Weekly phone check‐ins as needed. Txt msg 5 days/week.	Assess health risk behaviour and set change goals. Review progress and motivation.	NR | Treatment engagement (% participants responding to txt msg within first month) ‐ > 80% (n = 18) high response rate and n = 8 low treatment engagement.
Temmingh, 2013 [44]	The Wellness Program	One‐on‐one tele‐coaching sessions | Diet (meal plan), physical activity (exercise plan)	Telephone calls + e‐mail	N/A	Telecoaches ‐dieticians and biokineticists with motivational interview training via psychiatrist | Telephone	E‐mail: general health tips and healthy foods and recipes. Telephone calls: assess dietary habits and form a personal meal plan. Self‐monitoring of diet adherence.	12 months | 1 ×20‐30 min call with health behaviour assessment and meal plan provision. Weekly 5‐10 min calls to catch up on behaviour changes, self‐monitoring and reviewing goals for first 3 months, monthly calls for next 9 months.	Feedback on dietary and physical activity habits. Goal setting and self‐reflection.	Telecoaches attended initial training, with monthly quality assurance checks by team leader on random subsample of recorded calls. Monthly group and individual supervision sessions, retraining conducted if required. | Weekly self‐report by participants on adherence to meal and exercise plans (5‐point Likert scale, 1 = poor to 5 = excellent). Adherence to diet plans rated as good to excellent in 81.8% at 4 weeks, 81.4% at 8 weeks, and 86.3% at 12 weeks. Adherence to exercise plans rated as good to excellent in 50.2% at 4 weeks, 58.3% at 8 weeks, and 56.3%at 12 weeks.

Abbreviations: ↑ increase; ↓ decrease; CVD, cardiovascular disease; eHealth, electronic health; F&V, fruit and vegetables; MH, mental health; mHealth, mobile health; mins, minutes; N/A not assessed; NR, not reported; SSB, sugar sweetened beverages; TIDieR, Template for Intervention Description and Replication; txt msg, text message.

^a^ Items 2 and 10 of the TIDiER checklist not included. All published articles included the WHY (i.e. Item 2: Describe any rationale, theory, or goal of the elements essential to the intervention), and no studies reported any intervention modifications during the course of the study (i.e. Item 10: MODIFICATIONS).

All studies included both diet and physical activity/sedentary behaviour components in the intervention. The dietary components focused on increasing core foods, including increasing fruit and vegetables/healthy eating (*n* = 7 [34, 35, 36, 37, 39, 40, 41]), or had a weight loss (*n* = 5 [35, 36, 37, 38, 44]) or energy reduction (*n* = 1 [42]) focus. Fewer studies had components described for decreasing processed foods and/or sugar sweetened drinks (*n* = 3 [34, 35]). Other health behaviours were targeted in some interventions: two studies had a sleep component [34, 42], a further two studies included content around social connection [34, 36], and three studies included smoking reduction/substance use [39, 40, 41, 42]. Only two studies described having a mental health component/monitoring, however these were not described in detail [34, 41].

Various e‐health strategies were used across the included studies. Ten studies incorporated smartphone technology with five of these studies using phone calls [38, 39, 40, 41, 44], three studies sent text messages [35, 36, 37], one used a smartphone application [42], and one study was described broadly as multimodal [34]. Other online technologies were used in seven studies, consisting of private Facebook groups in three studies [35, 36, 37], web tool [43], wearable trackers in three studies [35, 36, 37], and email in one study [44]. Of the included studies, seven studies included face‐to‐face components alongside the technology [35, 36, 37, 38, 39, 40, 43], including group exercise sessions, education sessions, technology training, and consults to track progress. The remaining four studies solely consisted of technology components [34, 41, 42, 44]. Only three of the included studies applied theories of behaviour change. These included social learning theory [35], the transtheoretical model [42], goal‐setting theory [44], and social cognitive and self‐efficacy theory [44].

Participants were led by psychologists in three studies [39, 40, 41], mental health counselling/nurses in three studies [35, 37, 43], lifestyle health coaches in two studies [36, 38], and dietitians in two studies [37, 44]. One study was completely self‐directed [34], and one was run by adult mentors (peers) [42]. Nine studies reported tailoring the intervention, with eight of these studies personalised the intervention by adapting the education content to suit the individual and having the participants set their own goals for the intervention [36, 37, 38, 39, 40, 41, 43, 44]. The other study derived their intervention from an existing program and tailored the intervention to suit young adults [35].

### 3.5 Effectiveness of Interventions

The most common outcome assessed (Table 4, and Supporting Information S1: Table S3 and S4) across the included studies was anthropometrics (*n* = 8 studies), reported as change in body weight (*n* = 6 studies [35, 36, 37, 38, 42, 44]), BMI (*n* = 6 studies [35, 36, 37, 42, 43, 44]) and/or waist circumference (*n* = 4 studies [39, 42, 43, 44]). Overall, five [35, 36, 38, 42, 44] of the eight studies reported statistically significant improvements in body measurements. Regarding the direction of effect, positive intervention effects (i.e. change from baseline in pre‐post studies, change from baseline in the intervention group in RCTs) were reported on body weight in five studies [35, 36, 38, 42, 44], BMI in four studies [36, 42, 43, 44], and waist circumference in three studies [42, 43, 44]. Mean decreases in pre to post intervention body weight ranged from 0.8 to 4.8 kg with follow‐up timepoints ranging from 12 weeks to 12 months. Mean decreases in BMI ranged from 0.2 to 1.3 kg/m^2^ with follow‐up timepoints ranging from 12 weeks to 12 months, and mean decreases in waist circumference of 1.0–6.8 cm with follow‐up timepoints ranging from 28 weeks to 12 months. One study [37] reported there was no significant change in body weight or BMI from baseline, but the data was not shown to determine a direction of effect.

**Table 4:** Summary of outcomes.

			Positive change in outcome a | Statistically significant change in outcome b				
Author, year	Outcomes measured	Follow‐up timepoints from baseline	Dietary	Mental health	Anthro	CVD	Physical activity/Sedentary behaviour
Abbott, 2020 [34]	QoL, symptom burden, depressive symptoms	9–10 weeks	N/A	✓ | ✓	N/A	N/A	N/A
Aschbrenner, 2016a [36]	Body weight, BMI, cardiorespiratory fitness	6 months	N/A	N/A	✓ | ✓	N/A	✓ | x
Aschbrenner, 2016b [37]	Body weight, BMI, cardiorespiratory fitness	6 months	N/A	N/A	NR | x	N/A	NR | x
Aschbrenner, 2022 [35]	BMI, CVD risk reduction (5% or greater weight loss or ↑ > 50 metres on 6MWT), lipids (triglycerides, total cholesterol, HDL, LDL), HbA1c, BP, physical activity	6 and 12 months	N/A	N/A	✓ | ✓	✓ CVD risk, lipids | ✓ CVD risk	✓ | ✓
Baker, 2014 [41]	F&V intake and diet quality, depression scores, QoL, global functioning (GAF), screentime, physical activity	8 or 12 weeks (4 weeks post treatment)	✓ | ✓	✓ | ✓ QoL, GAF	N/A	N/A	✓ | ✓
Baker, 2015 [40]	CVD risk (ASSIGN score), Smoking status, F&V servings, psychiatric symptoms, global functioning (GAF), QoL, physical activity	15 weeks (mid‐intervention) and 12 months	✓ | x	✓ | ✓ depression, GAF	N/A	✓ | ✓	✓ | x
Baker, 2018 [39]	10‐year CVD risk, diet score (unhealthy eating index), depression scale, waist circumference, global functioning (GAF)	15 weeks (mid‐intervention), and 12, 18, 24, 30 and 36 months	x | x	✓ | ✓	✓ | x	✓ | ✓	✓ | x
Lee, 2020 [42]	Waist circumference, BMI, lipids (triglycerides, total cholesterol, HDL, LDL)	12 weeks and 28 weeks	N/A	N/A	✓ | ✓	✓ | ✓	N/A
Looijmans, 2019 [43]	Waist circumference (WC), BMI, metabolic syndrome z‐score	6 months (mid‐intervention) and 12 months	N/A	N/A	✓ WC | x	✓ | x	N/A
Nicol, 2022 [38]	Body weight	12 weeks	N/A	N/A	✓ | ✓	N/A	N/A
Temmingh, 2013 [44]	Body weight, waist circumference, BMI, general health (self‐rated scale)	12, 24, 36 and 48 weeks	N/A	✓ | ✓	✓ | ✓	N/A	N/A

Abbreviations: Anthro, anthropometric; BMI, body mass index; BP, blood pressure; CV, cardiovascular; CVD, cardiovascular disease; F&V, fruit and vegetable; HDL, high density lipoprotein cholesterol; LDL, low density lipoprotein cholesterol; N/A, not assessed; NR, data not reported; QoL, quality of life.

^a^ Tick indicates there was a positive improvement in the outcome assessed, and the change may or may not be statistically significant, compared with baseline at any stage of the intervention, that is, during the intervention, immediately post‐intervention, or at follow‐up; x indicates there was no improvement in the outcome assessed.

^b^ Tick indicates outcomes assessed and reported a statistically significant change (*p* < 0.05) compared with either the control or with baseline at any stage of the intervention, that is, during the intervention, immediately post‐intervention, or at follow‐up; x indicates the outcome assessed, but a significant change not reported.

The next most common outcomes in descending order were physical activity/sedentary behaviours assessed in six studies [35, 36, 37, 39, 40, 41], with two reporting significant improvements [35, 41]. Positive intervention effects were reported in four studies [35, 36, 40, 41]. This included mean increases in pre to post intervention walking time per week (ranging from 40 to 104 min/week across three follow‐up timepoints) in two studies [40, 41], increased distance on the 6‐min walk test ( ≥ 50 m at 6‐ and 12‐month follow‐ups) in two studies [35, 36], mean decreases in siting time (ranging from 27 to 294 min/week across three follow‐up timepoints) in two studies [40, 41] and mean decrease (294 and 27 min/week at 15 weeks and 12 months, respectively) in screen time in one study [41]. Mixed effects (i.e. increases and decreases in walking and sitting times per week), across six follow‐up timepoints (15 weeks, 12‐, 18‐, 24‐, 30‐ and 36‐months), was reported by one study [39]. Another study [37] reported there was no significant change in fitness from baseline, but the data was not shown to determine a direction of effect.

CVD outcomes were assessed in five studies [35, 39, 40, 42, 43], with three studies measuring changes in CVD risk [35, 39, 40], and three measuring changes in blood lipids [35, 42, 43]. While four [35, 39, 40, 42] of these studies reported significant improvements in CVD outcomes, all studies demonstrated positive intervention effects on CVD outcomes. Mental health outcomes were assessed in five studies [34, 39, 40, 41, 44]. This included depression and/or psychiatric symptomology in 4 studies [34, 39, 40, 41], QoL in four studies [34, 39, 40, 41], global functioning in three studies [39, 40, 41], and self‐rated general health in the remaining study [44]. All studies reported positive intervention effects on mental health outcomes with the majority (11 out of 13 outcomes) of these changes significant.

Dietary outcomes [diet quality (*n* = 2 [39, 41]) or fruit and vegetables subscales of diet quality (*n* = 2 [40, 41]) were the least common outcome assessed with only three studies [39, 40, 41] reporting on dietary changes. A significant increase in mean diet quality score (Australian Recommended Food Score) was reported in one pre‐post study [41] at 4 weeks post‐intervention [effect size −0.97 (95% CI; −1.48, −0.45)]. The other study that assessed diet quality [39], via a study specific unhealthy eating index, found minimal to no change in mean scores in the intervention or control groups at follow‐up timepoints of 15 weeks, 12‐, 18‐, 24‐, 30‐ and 36‐months (mean change in scores ranged from −0.5 to 0.0). Positive intervention effects were reported for fruit and vegetable intake with mean increases of 0.40 serves/day in two studies at 4 weeks post‐intervention [41] and at 12 months follow‐up [40].

### 3.6 Completeness of Intervention Reporting

The overall reporting of interventions shows a variation in the responses between several TIDieR citeria (Table 5). All 11 studies were 100% compliant with 6 of the 12 TIDieR criteria. This included naming within the study to describe the intervention, the rationale for the intervention, details of who provided the intervention and their expertise, how the intervention was delivered and whether it was delivered individually or in a group, where the intervention occurred, and when the intervention was delivered and over what period of time including the number of sessions. In 10 of the 11 interventions the procedures, activities, and/or processes used in the intervention were described well. One study reported basic intervention procedures only. Of the studies that provided tailoring of the interventions, the procedures used were described adequately in seven of the nine studies. Two studies provided no details on the variables/constructs that would be used to develop or assess participant's personalised plans or goals. The reporting of intervention fidelity criteria (i.e. planned and actual) was considered moderately compliant across the studies. Three studies reported either limited details relating to the fidelity process or the extent to which the delivered intervention varied from the intended intervention. Two studies did not report on any planned or actual fidelity procedures. One TIDieR criterion that was predominately not addressed adequately was ‘what materials’ were used in the intervention (physical or informational) or used in intervention delivery or in training of providers. No studies reported whether ‘modifications to the intervention’ were made during the course of the study.

**Table 5:** Summary of reporting quality of interventions as per TIDieR criteria checklist [30].

Author, year	Brief name	Why	What materials	What procedures	Who	How	Where	When	Tailoring	Modifications	How well planned	How well actual
Abbott, 2020 [34]	•	•	•	•	•	•	•	•	NA	•	•	•
Aschbrenner, 2016a [36]	•	•	•	•	•	•	•	•	•	•	•	•
Aschbrenner, 2016b [37]	•	•	•	•	•	•	•	•	•	•	•	•
Aschbrenner, 2022 [35]	•	•	•	•	•	•	•	•	•	•	•	•
Baker, 2014 [41]	•	•	•	•	•	•	•	•	•	•	•	•
Baker, 2015 [40]	•	•	•	•	•	•	•	•	•	•	•	•
Baker, 2018 [39]	•	•	•	•	•	•	•	•	•	•	•	•
Lee, 2020 [42]	•	•	•	•	•	•	•	•	NA	•	•	•
Looijmans, 2019 [43]	•	•	•	•	•	•	•	•	•	•	•	•
Nicol, 2022 [38]	•	•	•	•	•	•	•	•	•	•	•	•
Temmingh, 2013 [44]	•	•	•	•	•	•	•	•	•	•	•	•
Compliance (%)^a^	100	100	18	91	100	100	100	100	78	0	64	73

Abbreviation: TIDieR, Template for Intervention Description and Replication.

Key: • met the TIDieR requirements; • partially met the TIDieR requirements; • did not met the TIDieR requirements; or NA, not applicable/not planned.

^a^ Compliance indicates the percentage of studies who achieved a green rating.

## 4 Discussion

This review set out to synthesise the evidence for the effectiveness of e‐health delivered diet and nutritional interventions and outcomes in adults living with SMI. This review specifically evaluated interventions against the TIDieR framework to identify clinical directions for dietary e‐health interventions as well as evidence gaps. It was found that there is a limited body of evidence with only 11 studies included, and the most common intervention delivery format was a combination of e‐health and face‐to‐face. The overall risk of bias of studies was low with the majority of studies (8/11) rated as positive. The overall number of people in the studies was moderate, and there were relatively good retention rates (median 74%, range from 57% to 96%) at follow‐up in both males and females across studies. Study attrition can vary considerably in health behaviour change studies (ranging from 10% to 80% loss to follow‐up and/or dropouts) which can undermine the validity of findings [45, 46, 47]. Higher rates have been reported among vulnerable population groups [47] and in individuals with more severe mental health symptoms [48]. The overall retention rate found in this review suggests e‐health interventions are acceptable in SMI adult populations or e‐health modalities as an adjunct to traditionally delivered lifestyle interventions. The major results from the review indicate both shorter‐ and longer‐term e‐health interventions that focus on diet are effective in improving lifestyle behaviours that impact physical and mental health, as demonstrated by the number of positive outcomes in lifestyle behaviours.

The included studies involved interventions that aimed to address multiple lifestyle components and included a range of appropriate outcomes to measure physical health. However, anthropometrics, CVD, physical activity, and mental health outcomes more commonly assessed than diet. Given the inclusion criteria of the review was dietary focused interventions, and many involved weight management/weight loss components, it was expected that more studies would have included the assessment and reporting of dietary intakes in addition to the other physical health outcomes measured, along with greater dietetic involvement or interventions delivered by dietitians. Previous research in SMI have illustrated through meta‐analysis that dietary interventions run by dietitians are more effective for weight management and improving clinical outcomes than when compared to other delivery partners [10]. Given few studies in the current review were run by dietitians, it does highlight that dietary approaches in lifestyle interventions could be improved. For example, increased involvement of dietitians in the development and implementation of interventions and planning of outcomes measures using validated dietary assessment tools to enable the unique nutrition‐related challenges experienced by people living with SMI to be addressed. As accredited allied health practitioners, dietitians are well equipped to deliver Medical Nutrition Therapy via e‐health behaviour change interventions and are best placed to assist with major factors affecting physical health, such as nutritional inadequacy, disordered eating behaviours, metabolic side effects related to antipsychotic medication use and food insecurity or eating on a budget.

The most common e‐health modality used across studies was delivery of the intervention using smart phones or a telephone‐based method, rather than other e‐health technology such as computers, digital health platforms with telehealth options, video calls or apps which are increasingly used in health care delivery. Text messages were used in four studies [35, 36, 37, 38] and only one specifically using a study specific smart phone app [38]. The barriers experienced by individuals with SMI need to be considered when designing and implementing e‐health interventions. For example, individuals with low levels of digital literacy (such as a lack of basic computer skills, internet navigation abilities and lack of familiarity with smart phone technology) may find e‐health modalities challenging to engage with or navigate [20, 49]. Privacy and confidentiality concerns, including a lack of privacy in the physical setting and surroundings of the patient, have been raised [50, 51] and may also impact the adoption of e‐health modalities. A further consideration is the digital infrastructure of communities, particularly in remote and rural regions, to make full use of e‐health technology and ensure accessibility, reliability and security [52]. For example, access to reliable internet connectivity and secure servers to facilitate virtual consultations between patients and healthcare providers, availability of phone coverage, and access to affordable internet and phone plans [52]. Given the field of technology has evolved considerably, an opportunity exists to use and test feasibility of more features of digital/e‐health in dietary interventions for SMI. This could include components across the nutrition care process including dietary assessment, delivery of care through telehealth/video conferencing, reminders, compliance and outcome assessments. Further, the novel developments in dietary assessment with the use of image‐based assessments which are less reliant on memory and cognitive function [53, 54] may present opportunities to overcome barriers in SMI populations.

The importance of dietitians in mental health settings is increasingly recognised, however many public clinical services still do not have dietitians or have insufficient dietetic cover [12]. Private practice dietitians can potentially fill some of the gap left by public services, but this may not be financially viable for many individuals with SMI. Further evidence of the lack of dietitians is demonstrated by the fact that the majority of interventions in the included studies (*n* = 9/11) were delivered by health professionals or coaches who may or may not have specific training in dietary intakes, delivery of dietary based information and are not equipped to deliver Medical Nutrition Therapy. Also of importance is that reviews highlight the lack of dietary assessment tools validated for use in mentally ill populations [55]. In addition, a previous study of dietitians working in mental health services [12], reported that the most common dietary risk factor assessed in practice was malnutrition using the Malnutrition Screening Tool. While malnutrition can be a health concern for some individuals with SMI, it does not reflect other important issues such as increased energy intakes, increased weight status and physical health complications. All of which have been shown to be prevalent issues and more of a concern in individuals with SMI [7], indicating other dietary assessment tools may be more suited. Given the importance of dietary intakes as a key modifiable risk factor, future studies for e‐health interventions should include a measure of diet.

A strength of the current review is that the dietary interventions of the included studies were mapped to the TIDieR criteria. This enabled a more comprehensive assessment of the interventions than the validity questions related to the intervention description in the risk of bias tool allows. The TIDieR framework aims to promote transparency and improve the quality of reporting, to facilitate better translation of research into practice. Results from the included studies in the review show that many dietary interventions reported tailoring of the interventions for participants, but few interventions were modelled on behaviour change theories. This is interesting given the outcomes of many studies were behaviour change focused. A specific focus was weight loss in five studies [35, 36, 37, 38, 44] and the majority centred around improved core foods/diet quality. It was noted in one study [37] where the major focus was on calorie balance that specific adaptations may be needed for optimal weight management treatment in individuals with SMI. For example, focusing on eating a balanced diet (e.g., increasing fruit and vegetable consumption) rather than a caloric restriction diet as this may be more sustainable in daily life. Dietary interventions using e‐health modalities are recommended to be modelled on behaviour change theories in future as this may aid in improving their outcomes. A reporting recommendation arising from this review includes documenting actual intervention fidelity in future studies, as the majority of studies (*n* = 8) reported on participants who remained in the study, rather than capturing the adherence to the intervention (i.e. number of treatments received by each participant) or engagement with the intervention in the case of self‐directed online interventions or apps. Attrition should not be used as a proxy for actual intervention fidelity, as participants may remain in a study and provide outcome data, whilst not receiving all the treatments as planned. This has potential to affect study findings. It should also be noted that in two of the included studies, researchers calculated changes in BMI using self‐reported height and weight data. Similar to findings in the general population, previous research in individuals with SMI with overweight/obesity show small overestimations in height (1.0 ± 2.9 cm in SMI [56] vs 1.4 ± 1.9 cm in the general population [57]) and small underestimations in weight (0.1 ± 5.6 kg in SMI [56] vs 0.6 ± 2.0 kg in the general population [57]) can occur with self‐reported anthropometric data. Although there is potential for reporting bias, previous research indicates that online self‐reported height and weight can be accepted as a satisfactory method of data collection in web‐based interventions [57].

A previous review of e‐health behaviour change interventions in SMI [22] demonstrated that for those interventions focused on physical health; such as cardiometabolic, weight loss, and physical activity outcomes; the highest recruitment rates were observed in studies conducted through outpatient clinics with subsequent participation not involving additional in‐person sessions. Usage of digital interventions is high whereby previous studies reporting better completion or more modules completed by those in the digital arm compared to the in‐person arm [58].

In the field of SMI, terminology in practice and in guidelines [59] often refer to ‘lifestyle interventions’ or ‘health behaviour change interventions’ with these often being recommended, however this is a broad term and defined and implemented in different ways in research. Consistent with clinical practice guidelines for mood disorders [59], a previous review by Sawyer et al. [22] found that the majority of health behaviour change interventions in SMI were focused on smoking cessation, physical activity and/or cardio‐metabolic health, and a small number (3/36 included studies) concerned other health behaviours. It is timely to consider dietary approaches in lifestyle interventions as diet is a key modifiable risk factor for improved physical and mental health in those with SMI. Regarding dietary components of interventions, in the current review the majority of studies primarily focused on promoting core foods with fewer focusing on processed or discretionary food choices. Interestingly six of the included studies had weight loss or an energy restriction focus, however these were not often implemented by health professionals but instead by lifestyle coaches.

The results of this review are consistent with previous reviews in that digital interventions are acceptable to people with an SMI [15, 22], which can present a promising option for addressing behavioural health in these populations. Specifically, for those in rural or regional areas where this technology provides opportunity by increasing access to care. Previous studies suggest that additional human support may be useful for promoting adherence/engagement, and the content of such interventions may benefit from more tailoring to specific needs [16, 60, 61]. While the literature does not yet allow for conclusions regarding efficacy for mental health, the available evidence to date does support their potential to change behaviour across various domains. However as evidenced by this review, dietary interventions via e‐health are not common.

The major strengths of this review are the use of a comprehensive search strategy and screening process, as well as the use of a standardised risk of bias tool to assess study quality. Further, the review advances nutritional science by use of the TIDieR framework to identify research opportunities to inform clinical service [30]. Due to the diversity and heterogeneity of quantitative outcomes reported and the measures in which they were collected a meta‐analysis was not possible which may limit the generalisability of our conclusion. The generalisability of the findings may also be limited due to the small number of included studies and overall low number of participants. While the risk of bias assessment indicated a low level of risk for outcomes within studies, the certainty of the evidence was not assessed using a formal framework e.g. GRADE framework (Grading of Recommendations, Assessment, Development, and Evaluations) [62]. Therefore, the results should be viewed in light of this. Further limitations of this review include that studies were restricted to only those published in English, which may have excluded some relevant evidence in other languages. This review included only those studies which included treatment interventions for those with a mental disorder diagnosis. Therefore, studies of interventions aiming to prevent mental disorders or including individuals without a mental disorder diagnosis were excluded as this was outside the scope of the review. This may have excluded some relevant evidence, however to include these would have affected the specificity of the review findings.

## 5 Conclusion

The findings from this review synthesise findings of the evidence base for the use of e‐health for dietary interventions in individuals with SMI. There is large scope to harness e‐health technologies further across the nutrition care process such as assessment, delivery of interventions, and reminders, and also technology‐based platforms to assess outcomes. Future dietary interventions should include assessment of dietary intakes to determine the resultant changes from dietary focused interventions and make use of all TIDieR framework criteria to improve reporting of interventions to further the field.

## Author Contributions

K.H., S.H. and T.B. conceptualised the review. K.H., S.H., J.S. and T.B. screened references, extracted data and completed quality assessment, analysed and interpreted the data. A.S. assisted in search strategy development and completed literature searches. All authors contributed to the writing and reviewing of the manuscript. All authors have read and approved the final version submitted for publication.

## Conflicts of Interest

The authors declare no conflicts of interest.

## 1 Peer Review

The peer review history for this article is available at https://www.webofscience.com/api/gateway/wos/peer-review/10.1111/jhn.70112.

## Supporting information

Supplementary material.

## Data Availability

The data that support the findings of this study are openly available in the following electronic databases: Cochrane Library at https://www.cochranelibrary.com/, PsycInfo at https://www.apa.org/pubs/databases/psycinfo/, CINAHL at https://www.ebsco.com/products/research-databases/cinahl-database, MEDLINE at https://www.nlm.nih.gov/medline/medline_home.html, or Embase at https://www.embase.com/.
