# Supplementary material for: Dietary E‐Health Interventions for Adults With Severe Mental Illness: A Systematic Review
Source: J Hum Nutr Diet. 2025 Aug 24;38(4):e70112. doi: 10.1111/jhn.70112 (PMC12375891; doi:10.1111/jhn.70112)
Supplement: Supplementary file 1 — Supplementary material. [file JHN-38-0-s001.pdf]

PRISMA 2020 Checklist

Section and Topic	Item #	Checklist item	Location where item is reported
**TITLE**			
Title	1	Identify the report as a systematic review.	Title
**ABSTRACT**			
Abstract	2	See the PRISMA 2020 for Abstracts checklist.	Page 1-2
**INTRODUCTION**			
Rationale	3	Describe the rationale for the review in the context of existing knowledge.	Lines 31-97
Objectives	4	Provide an explicit statement of the objective(s) or question(s) the review addresses.	Lines 99-107
**METHODS**			
Eligibility criteria	5	Specify the inclusion and exclusion criteria for the review and how studies were grouped for the syntheses.	Table 1, Lines 128-136
Information sources	6	Specify all databases, registers, websites, organisations, reference lists and other sources searched or consulted to identify studies. Specify the date when each source was last searched or consulted.	Lines 115-126
Search strategy	7	Present the full search strategies for all databases, registers and websites, including any filters and limits used.	Supplementary material
Selection process	8	Specify the methods used to decide whether a study met the inclusion criteria of the review, including how many reviewers screened each record and each report retrieved, whether they worked independently, and if applicable, details of automation tools used in the process.	Table 1<br>Lines 115-126
Data collection process	9	Specify the methods used to collect data from reports, including how many reviewers collected data from each report, whether they worked independently, any processes for obtaining or confirming data from study investigators, and if applicable, details of automation tools used in the process.	Lines 138-144, 166-175
Data items	10a	List and define all outcomes for which data were sought. Specify whether all results that were compatible with each outcome domain in each study were sought (e.g. for all measures, time points, analyses), and if not, the methods used to decide which results to collect.	Lines 157-179
	10b	List and define all other variables for which data were sought (e.g. participant and intervention characteristics, funding sources). Describe any assumptions made about any missing or unclear information.	Lines 157-179
Study risk of bias assessment	11	Specify the methods used to assess risk of bias in the included studies, including details of the tool(s) used, how many reviewers assessed each study and whether they worked independently, and if applicable, details of automation tools used in the process.	Lines 138-152
Effect measures	12	Specify for each outcome the effect measure(s) (e.g. risk ratio, mean difference) used in the synthesis or presentation of results.	NA
Synthesis methods	13a	Describe the processes used to decide which studies were eligible for each synthesis (e.g. tabulating the study intervention characteristics and comparing against the planned groups for each synthesis (item #5)).	Tables 1 and 2
	13b	Describe any methods required to prepare the data for presentation or synthesis, such as handling of missing summary statistics, or data conversions.	NA
	13c	Describe any methods used to tabulate or visually display results of individual studies and syntheses.	Lines 164 and 178-179
	13d	Describe any methods used to synthesize results and provide a rationale for the choice(s). If meta-analysis was performed, describe the model(s), method(s) to identify the presence and extent of statistical heterogeneity, and software package(s) used.	NR – no meta-analysis
	13e	Describe any methods used to explore possible causes of heterogeneity among study results (e.g. subgroup analysis, meta-regression).	NR
	13f	Describe any sensitivity analyses conducted to assess robustness of the synthesized results.	NR
Reporting bias	14	Describe any methods used to assess risk of bias due to missing results in a synthesis (arising from reporting biases).	Supplementary

PRISMA 2020 Checklist

Section and Topic	Item #	Checklist item	Location where item is reported
assessment			material
Certainty assessment	15	Describe any methods used to assess certainty (or confidence) in the body of evidence for an outcome.	NA
**RESULTS**			
Study selection	16a	Describe the results of the search and selection process, from the number of records identified in the search to the number of studies included in the review, ideally using a flow diagram.	Figure 1
	16b	Cite studies that might appear to meet the inclusion criteria, but which were excluded, and explain why they were excluded.	Figure 1
Study characteristics	17	Cite each included study and present its characteristics.	Lines 182-266 and 319-338<br>Tables 2, 3, and 5
Risk of bias in studies	18	Present assessments of risk of bias for each included study.	Supplementary table S2
Results of individual studies	19	For all outcomes, present, for each study: (a) summary statistics for each group (where appropriate) and (b) an effect estimate and its precision (e.g. confidence/credible interval), ideally using structured tables or plots.	Lines 268- 317<br>Table 3, 4 and 5<br>Supplementary table S3 and S4
Results of syntheses	20a	For each synthesis, briefly summarise the characteristics and risk of bias among contributing studies.	Lines 217-225
	20b	Present results of all statistical syntheses conducted. If meta-analysis was done, present for each the summary estimate and its precision (e.g. confidence/credible interval) and measures of statistical heterogeneity. If comparing groups, describe the direction of the effect.	No meta-analysis
	20c	Present results of all investigations of possible causes of heterogeneity among study results.	Lines 194-266
	20d	Present results of all sensitivity analyses conducted to assess the robustness of the synthesized results.	NR
Reporting biases	21	Present assessments of risk of bias due to missing results (arising from reporting biases) for each synthesis assessed.	Supplementary table S2
Certainty of evidence	22	Present assessments of certainty (or confidence) in the body of evidence for each outcome assessed.	Tables 2, 3, 4 and 5
**DISCUSSION**			
Discussion	23a	Provide a general interpretation of the results in the context of other evidence.	Lines 341-483
	23b	Discuss any limitations of the evidence included in the review.	Lines 488-492
	23c	Discuss any limitations of the review processes used.	Lines 492-502
	23d	Discuss implications of the results for practice, policy, and future research.	Lines 505-511
**OTHER INFORMATION**			
Registration and protocol	24a	Provide registration information for the review, including register name and registration number, or state that the review was not registered.	Lines 110-111
	24b	Indicate where the review protocol can be accessed, or state that a protocol was not prepared.	Lines 110-111

PRISMA 2020 Checklist

Section and Topic	Item #	Checklist item	Location where item is reported
	24c	Describe and explain any amendments to information provided at registration or in the protocol.	NA
Support	25	Describe sources of financial or non-financial support for the review, and the role of the funders or sponsors in the review.	Title page
Competing interests	26	Declare any competing interests of review authors.	Title page
Availability of data, code and other materials	27	Report which of the following are publicly available and where they can be found: template data collection forms; data extracted from included studies; data used for all analyses; analytic code; any other materials used in the review.	NA

From: Page MJ, McKenzie JE, Bossuyt PM, Boutron I, Hoffmann TC, Mulrow CD, et al. The PRISMA 2020 statement: an updated guideline for reporting systematic reviews. BMJ 2021;372:n71. doi: 10.1136/bmj.n71

For more information, visit: http://www.prisma-statement.org/

Supplementary Table S1. Search strategy

Database: Ovid MEDLINE(R) and Epub Ahead of Print, In-Process, In-Data-Review & Other Non-Indexed Citations and Daily <1946 to June 20, 2023>
Search Strategy:

1 exp "Schizophrenia Spectrum and Other Psychotic Disorders"/ (162776)
2 Bipolar Disorder/ (45113)
3 exp Depressive Disorder/ (121800)
4 ((depression or depressive) adj2 (disorder* or severe or major or treatment resistant or endogenous)).ti,ab,kw,kf. (93868)
5 ((severe or serious) adj (mental illness* or mental health or mental disorder* or psychiatric or psychologic*)).ti,ab,kw,kf. (17993)
6 (psychotic or psychoses or psychosis).ti,ab,kw,kf. (82000)
7 (schizoaffective or schizo-affective or schizophreni*).ti,ab,kw,kf. (144666)
8 (bipolar depression or bipolar disorder* or bi-polar depression or bi-polar disorder* or unipolar depression or unipolar disorder* or uni-polar depression or uni-polar disorder*).ti,ab,kw,kf. (38637)
9 dysthymi*.ti,ab,kw,kf. (3332)
10 1 or 2 or 3 or 4 or 5 or 6 or 7 or 8 or 9 (415378)
11 Feeding Behavior/ (92594)
12 Eating/ (59399)
13 (food intake* or food habit* or nutrition*).ti,ab,kw,kf. (412936)
14 (diet* adj5 (intake* or behavio?r* or quality or habit* or pattern* or guideline*)).ti,ab,kw,kf. (120467)
15 (eat* adj3 (behavio?r* or pattern* or habit*)).ti,ab,kw,kf. (28174)
16 (fruit* or vegetable* or fat or fats or fiber* or fibre* or sugar* or energy dense food* or sometimes food* or discretionary food* or core food* or Mediterranean).ti,ab,kw,kf. (1051190)
17 Life Style/ (63615)
18 (life style or lifestyle).ti,ab,kw,kf. (134632)
19 11 or 12 or 13 or 14 or 15 or 16 or 17 or 18 (1650724)
20 Telemedicine/ (37250)
21 Remote Consultation/ (5723)
22 Videoconferencing/ (2319)
23 (telemed* or tele-med* or telehealth or tele-health or telecare or tele-care* or tele-psychiatr* or telepsychiatr* or telepsychogeriatric* or tele-psychogeriatric* or telegeriatric* or tele-geriatric* or telemental or tele-mental or teleneuropsycholog* or tele-neuropsycholog* or tele-behavio?r* or telebehavio?r*).ti,ab,kw,kf. (36229)
24 (teleassess* or tele-assess* or teleassist* or tele-assist* or telebased or tele-based).ti,ab,kw,kf. (193)
25 (video* or teleconsult* or tele-consult* or virtual provider* or virtual appointment* or virtual consult* or virtual visit* or skype or facetime or mobile health or mhealth or ehealth or e-health or e-consult* or econsult*).ti,ab,kw,kf. (193493)
26 (remote adj2 (consult* or diagnos* or monitor* or assess*)).ti,ab,kw,kf. (8638)
27 (iphone* or i-phone* or smartphone* or smart phone* or ipad* or i-pad* or PDA* or personal digital assitant* or hand-held device* or handheld device* or mobile device* or

mobile technolog* or mobile tablet* or laptop* or wi-fi or wifi).ti,ab,kw,kf. (67182)
28 (ehealth or e-health or etherap* or e-therap* or emental or e-mental or electronic health or electronic technolog*).ti,ab,kw,kf. (42521)
29 (internet adj (based or enabl* or mediated or supported or deliver*)).ti,ab,kw,kf. (12252)
30 (web-based or web based or webpage* or web page* or website* or web site* or web app* or webapp* or online or on-line or computer based or computer-based).ti,ab,kw,kf. (330067)
31 digital intervention*.ti,ab,kw,kf. (1523)
32 20 or 21 or 22 or 23 or 24 or 25 or 26 or 27 or 28 or 29 or 30 or 31 (636583)
33 10 and 19 and 32 (306)
34 limit 33 to english language (295)
35 limit 34 to (comment or editorial or letter or news) (2)
36 34 not 35 (293)

Database: Embase <1974 to 2023 June 20>
Search Strategy:
1 exp schizophrenia spectrum disorder/ (210740)
2 exp bipolar disorder/ (78228)
3 depressive psychosis/ or major depression/ or treatment resistant depression/ or endogenous depression/ or dysthymia/ (93545)
4 ((depression or depressive) adj2 (disorder* or severe or major or treatment resistant or endogenous)).ti,ab,kw,kf. (133572)
5 ((severe or serious) adj (mental illness* or mental health or mental disorder* or psychiatric or psychologic*)).ti,ab,kw,kf. (24169)
6 (psychotic or psychoses or psychosis).ti,ab,kw,kf. (113521)
7 (schizoaffective or schizo-affective or schizophreni*).ti,ab,kw,kf. (193400)
8 (bipolar depression or bipolar disorder* or bi-polar depression or bi-polar disorder* or unipolar depression or unipolar disorder* or uni-polar depression or uni-polar disorder*).ti,ab,kw,kf. (61407)
9 dysthymi*.ti,ab,kw,kf. (4416)
10 1 or 2 or 3 or 4 or 5 or 6 or 7 or 8 or 9 (492559)
11 feeding behavior/ (93885)
12 eating/ or food intake/ (189081)
13 (food intake* or food habit* or nutrition*).ti,ab,kw,kf. (534207)
14 (diet* adj5 (intake* or behavio?r* or quality or habit* or pattern* or guideline*)).ti,ab,kw,kf. (156220)
15 (eat* adj3 (behavio?r* or pattern* or habit*)).ti,ab,kw,kf. (38813)
16 (fruit* or vegetable* or fat or fats or fiber* or fibre* or sugar* or energy dense food* or sometimes food* or discretionary food* or core food* or Mediterranean).ti,ab,kw,kf. (1249536)
17 lifestyle/ (137569)
18 (life style or lifestyle).ti,ab,kw,kf. (192265)
19 11 or 12 or 13 or 14 or 15 or 16 or 17 or 18 (2066840)
20 exp telehealth/ (87143)

21 videoconferencing/ (8895)
22 (telemed* or tele-med* or telehealth or tele-health or telecare or tele-care* or tele-psychiatr* or telepsychiatr* or telepsychogeriatric* or tele-psychogeriatric* or telegeriatric* or tele-geriatric* or telemental or tele-mental or teleneuropsycholog* or tele-neuropsycholog* or tele-behavio?r* or telebehavio?r*).ti,ab,kw,kf. (49707)
23 (teleassess* or tele-assess* or teleassist* or tele-assist* or telebased or tele-based).ti,ab,kw,kf. (275)
24 (video* or teleconsult* or tele-consult* or virtual provider* or virtual appointment* or virtual consult* or virtual visit* or skype or facetime or mobile health or mhealth or ehealth or e-health or e-consult* or econsult*).ti,ab,kw,kf. (272340)
25 (remote adj2 (consult* or diagnos* or monitor* or assess*)).ti,ab,kw,kf. (13486)
26 (iphone* or i-phone* or smartphone* or smart phone* or ipad* or i-pad* or PDA* or personal digital assitant* or hand-held device* or handheld device* or mobile device* or mobile technolog* or mobile tablet* or laptop* or wi-fi or wifi).ti,ab,kw,kf. (97452)
27 (ehealth or e-health or etherap* or e-therap* or emental or e-mental or electronic health or electronic technolog*).ti,ab,kw,kf. (59910)
28 (internet adj (based or enabl* or mediated or supported or deliver*)).ti,ab,kw,kf. (16209)
29 (web-based or web based or webpage* or web page* or website* or web site* or web app* or webapp* or online or on-line or computer based or computer-based).ti,ab,kw,kf. (455306)
30 digital intervention*.ti,ab,kw,kf. (1559)
31 20 or 21 or 22 or 23 or 24 or 25 or 26 or 27 or 28 or 29 or 30 (908033)
32 10 and 19 and 31 (475)
33 limit 32 to english language (466)
34 limit 33 to (books or chapter or conference abstract or conference paper or "conference review" or editorial or letter or note) (130)
35 33 not 34 (336)

Database: APA PsycInfo <1806 to June Week 2 2023>
Search Strategy:
1 serious mental illness/ (6429)
2 exp schizophrenia/ (98961)
3 exp psychosis/ (128128)
4 exp bipolar disorder/ (34371)
5 exp major depression/ (158539)
6 ((depression or depressive) adj2 (disorder* or severe or major or treatment resistant or endogenous)).ti,ab,id. (84065)
7 ((severe or serious) adj (mental illness* or mental health or mental disorder* or psychiatric or psychologic*)).ti,ab,id. (18296)
8 (psychotic or psychoses or psychosis).ti,ab,id. (87156)
9 (schizoaffective or schizo-affective or schizophreni*).ti,ab,id. (135091)
10 (bipolar depression or bipolar disorder* or bi-polar depression or bi-polar disorder* or unipolar depression or unipolar disorder* or uni-polar depression or uni-polar disorder*).ti,ab,id. (35830)

11 dysthymi*.ti,ab,id. (3980)
12 1 or 2 or 3 or 4 or 5 or 6 or 7 or 8 or 9 or 10 or 11 (387659)
13 eating behavior/ (16610)
14 food intake/ (15621)
15 (food intake* or food habit* or nutrition*).ti,ab,id. (42240)
16 (diet* adj5 (intake* or behavio?r* or quality or habit* or pattern* or guideline*)).ti,ab,id. (15032)
17 (eat* adj3 (behavio?r* or pattern* or habit*)).ti,ab,id. (17915)
18 (fruit* or vegetable* or fat or fats or fiber* or fibre* or sugar* or energy dense food* or sometimes food* or discretionary food* or core food* or Mediterranean).ti,ab,id. (65164)
19 lifestyle/ (12191)
20 (life style or lifestyle).ti,ab,id. (33441)
21 13 or 14 or 15 or 16 or 17 or 18 or 19 or 20 (158852)
22 exp telemedicine/ (13077)
23 (telemed* or tele-med* or telehealth or tele-health or telecare or tele-care* or tele-psychiatr* or telepsychiatr* or telepsychogeriatric* or tele-psychogeriatric* or telegeriatric* or tele-geriatric* or telemental or tele-mental or teleneuropsycholog* or tele-neuropsycholog* or tele-behavio?r* or telebehavio?r*).ti,ab,id. (7411)
24 (teleassess* or tele-assess* or teleassist* or tele-assist* or telebased or tele-based).ti,ab,id. (60)
25 (video* or teleconsult* or tele-consult* or virtual provider* or virtual appointment* or virtual consult* or virtual visit* or skype or facetime or mobile health or mhealth or ehealth or e-health or e-consult* or econsult*).ti,ab,id. (81958)
26 (remote adj2 (consult* or diagnos* or monitor* or assess*)).ti,ab,id. (786)
27 (iphone* or i-phone* or smartphone* or smart phone* or ipad* or i-pad* or PDA* or personal digital assitant* or hand-held device* or handheld device* or mobile device* or mobile technolog* or mobile tablet* or laptop* or wi-fi or wifi).ti,ab,id. (15493)
28 (ehealth or e-health or etherap* or e-therap* or emental or e-mental or electronic health or electronic technolog*).ti,ab,id. (6667)
29 (internet adj (based or enabl* or mediated or supported or deliver*)).ti,ab,id. (7261)
30 (web-based or web based or webpage* or web page* or website* or web site* or web app* or webapp* or online or on-line or computer based or computer-based).ti,ab,id. (162259)
31 digital intervention*.ti,ab,id. (468)
32 22 or 23 or 24 or 25 or 26 or 27 or 28 or 29 or 30 or 31 (259401)
33 12 and 21 and 32 (281)
34 limit 33 to english language (265)
35 limit 34 to ("0200 book" or "0240 authored book" or "0280 edited book" or "0300 encyclopedia" or "0400 dissertation abstract") (29)
36 34 not 35 (236)

Database: Cochrane Library

ID	Search Hits
#1	MeSH descriptor: [Schizophrenia Spectrum and Other Psychotic Disorders] explode all trees	12113
#2	MeSH descriptor: [Bipolar Disorder] this term only	3603
#3	MeSH descriptor: [Depressive Disorder] explode all trees	15098
#4	((depression or depressive) Near/2 (disorder* or severe or major or "treatment resistant" or endogenous)):ti,ab	20074
#5	((severe or serious) Next ("mental illness" or "mental illnesses" or mental health or "mental disorder" or "mental disorders" or psychiatric or psychologic*)):ti,ab	3758
#6	(psychotic or psychoses or psychosis):ti,ab	9376
#7	(schizoaffective or schizo-affective or schizophreni*):ti,ab	18342
#8	((bipolar or bi-polar or unipolar or uni-polar) NEXT (depression or disorder*)):ti,ab	5540
#9	dysthymi*:ti,ab	673
#10	#1 or #2 or #3 or #4 or #5 or #6 or #7 or #8 or #9	56184
#11	MeSH descriptor: [Feeding Behavior] this term only4264
#12	MeSH descriptor: [Eating] this term only	3668
#13	(food NEXT (intake* or habit*)):ti,ab	6449
#14	nutrition*:ti,ab42956
#15	(diet* Near/5 (intake* or behavio?r* or quality or habit* or pattern* or guideline*)):ti,ab	20874
#16	(eat* Near/3 (behavio?r* or pattern* or habit*)):ti,ab	4785
#17	(("energy dense" or sometimes or discretionary or core) NEXT food*):ti,ab	347
#18	(fruit* or vegetable* or fat or fats or fiber* or fibre* or sugar* or Mediterranean):ti,ab	69936
#19	MeSH descriptor: [Life Style] this term only 4404
#20	(life NEXT style or lifestyle):ti,ab	24253
#21	#11 or #12 or #13 or #14 or #15 or #16 or #17 or #18 or #19 or #20136861
#22	MeSH descriptor: [Telemedicine] this term only	3538
#23	MeSH descriptor: [Remote Consultation] this term only	415
#24	MeSH descriptor: [Videoconferencing] this term only	331
#25	(telemed* or tele-med* or telehealth or tele-health or telecare or tele-care* or tele-psychiatr* or telepsychiatr* or telepsychogeriatric* or tele-psychogeriatric* or telegeriatric* or tele-geriatric* or telemental or tele-mental or teleneuropsycholog* or tele-neuropsycholog* or tele-behavio?r* or telebehavio?r*):ti,ab	4798
#26	(teleassess* or tele-assess* or teleassist* or tele-assist* or telebased or tele-based):ti,ab	72
#27	(video* or teleconsult* or tele-consult* or skype or facetime or mhealth or ehealth or e-health or e-consult* or econsult*):ti,ab	32173
#28	(virtual NEXT (provider* or appointment* or consult* or visit*)):ti,ab	185
#29	(mobile NEXT health):ti,ab	1923
#30	(remote Near/2 (consult* or diagnos* or monitor* or assess*)):ti,ab1474
#31	(iphone* or i-phone*):ti,ab	346

#32 (smartphone* or smart NEXT phone*):ti,ab 7567
#33 (ipad* or i-pad*):ti,ab 919
#34 (PDA or PDAs or "personal digital assitant" or "personal digital assistants"):ti,ab 1274
#35 (("hand held" or hand-held or handheld) NEXT device*):ti,ab 297
#36 (mobile NEXT (device* or technolog* or tablet*)):ti,ab 1541
#37 (laptop* or wi-fi or wifi):ti,ab 904
#38 (ehealth or e-health or etherap* or e-therap* or emental or e-mental):ti,ab 2055
#39 (electronic NEXT (health or technolog*)):ti,ab 2513
#40 (internet NEXT (based or enabl* or mediated or supported or deliver*)):ti,ab 5034
#41 (web-based or "web based" or webpage or webpages or "web page" or "web pages" or website* or "web site" or "web sites" or "web app" or "web apps" or webapp or webapps or online or on-line or "computer based" or computer-based):ti,ab 38446
#42 ("digital intervention" or "digital interventions"):ti,ab 528
#43 #22 or #23 or #24 or #25 or #26 or #27 or #28 or #29 or #30 or #31 or #32 or #33 or #34 or #35 or #36 or #37 or #38 or #39 or #40 or #41 or #42 84385
#44 #10 and #21 and #43 215

Supplementary Table S2. Quality of included studies

Study quality and risk of bias assessed using ©2016 Evidence Analysis Manual Academy of Nutrition and Dietetics. Available from: https://www.andeal.org/vault/2440/web/files/2016_April_EA_Manual.pdf

	Relevance Questions							Validity Questions							
	R1	R2	R3	R4	V1	V2	V3	V4	V5	V6	V7	V8	V9	V10	
**Author, year, country**	**Improved outcomes for the patients/ clients/ population group**	**Outcome/s that patients/ clients/ population group would care about**	**Study topic relevant to dietetic practice**	**Feasible intervention procedure**	**Research question clearly stated**	**Participant selection free from bias**	**Study groups comparable**	**Withdrawals described**	**Blinding of outcomes and risk factors**	**Intervention described**	**Outcomes defined, measures valid**	**Appropriate statistical analysis**	**Conclusions supported**	**Funding bias unlikely**	**OVERALL rating***
Abbott, 2020, USA[1]	Y	Y	Y	Y	Y	Y	UC	Y	N	Y	Y	Y	Y	N	Ø
Aschbrenner, 2016a, USA[2]	Y	Y	Y	Y	Y	Y	N/A	Y	N/A	Y	Y	Y	Y	Y	+
Aschbrenner, 2016b, USA[3]	Y	Y	Y	Y	Y	Y	N/A	Y	N/A	Y	Y	Y	Y	Y	+
Aschbrenner, 2022, USA[4]	Y	Y	Y	Y	Y	Y	Y	Y	Y	Y	Y	Y	Y	Y	+
Baker, 2014, Australia[5]	Y	Y	Y	Y	Y	Y	N/A	Y	N/A	Y	Y	Y	Y	Y	+
Baker, 2015, Australia[6]	Y	Y	Y	Y	Y	Y	Y	Y	Y	Y	Y	Y	Y	Y	+
Baker, 2018, Australia[7]	Y	Y	Y	Y	Y	Y	Y	Y	Y	Y	Y	Y	Y	Y	+
Lee, 2020, South Korea[8]	Y	Y	Y	Y	Y	UC	N/A	Y	N/A	Y	Y	Y	Y	Y	Ø
Looijmans, 2019, Netherlands[9]	Y	Y	Y	Y	Y	Y	N	Y	Y	Y	Y	Y	Y	Y	Ø
Nicol, 2022, USA[10]	Y	Y	Y	Y	Y	Y	N/A	Y	N/A	Y	Y	Y	Y	Y	+

Temmingh, 2013, South Africa[11]	Y	Y	Y	Y	Y	Y	N/A	Y	N/A	Y	UC	Y	Y	N	Ø

N/A, not applicable, Y, yes, N, no, UC, unclear

*Overall quality ratings

MINUS/NEGATIVE (-) If most (six or more) of the answers to the above validity questions are “No,” the report is with a minus (-) symbol on the Evidence Worksheet. NEUTRAL (Ø) If the answers to validity criteria questions 2, 3, 6, and 7 do not indicate that the study is exceptionally strong, the report is designated with a neutral (Ø) symbol on the Evidence Worksheet.
PLUS/POSITIVE (+) If most of the answers to the above validity questions are “Yes” (including criteria 2, 3, 6, 7 and at least one additional “Yes”), the report is designated with a plus symbol (+) on the Evidence Worksheet.

Supplementary Table S3. Summary of outcomes

Author, year	Follow-up timepoints from baseline	Lifestyle Outcomes	Positive change in outcome [a] (Change from baseline in pre-post studies, or change from baseline in the intervention group in RCTs)	Statistically significant change in outcome [b] (Change from baseline in pre-post studies, or between group differences in RCTs)
Abbott, 2020[1]	9-10 weeks	Median of differences (IQR) in pre and post scores<br><br>**Mental health**<br>*Intervention group*:<br>PHQ-9<br>Total score -3 (8.25), p<0.001<br>MSQ<br>Total score -36 (31), p<0.001<br>SF-36<br>Physical function = 5 (10), p=0.024<br>Physical role functioning = 0 (50), p=0.971<br>Emotional role functioning = 0 (70), p=0.122<br>Vitality = 20 (30), p<0.001<br>Mental health = 22 (32), p<0.001<br>Sole role functioning =13 (31), p=0.060<br>Bodily pain = 13 (35), p=0.003<br>General health = 10 (26), p=0.003<br>*Control group*:<br>NS for all outcomes (all p >0.05)	✓	✓
Aschbrenner, 2016a[2]	6 months	Mean ± SD change<br><br>**Anthro**<br>Body weight = -7.8 ± 12.4 lbs, p=0.005.<br>BMI = -1.3 ± 2.0 kg/m^2, p = 0.005	✓	✓

		**Physical activity/ Sedentary behaviour**<br>6-MWT = 74.8 ± 205.1 feet, p = 0.09	✓	x
Aschbrenner, 2016b[3]	6 months	**Anthro**<br>NS change in body weight (data not reported)	Data not reported	x
		**Physical activity/Sedentary behaviour**<br>NS change in 6-MWT (data not reported)	Data not reported	x
Aschbrenner, 2022[4]	6 and 12 months	Between-group difference: group intervention (PeerFIT) vs one-on-one intervention (BEAT) mean ± SD scores at baseline, 6 and 12 months<br><br>Within-group difference at 6 and 12 months		
		**Anthro**<br>Between-group difference<br>Weight (kg): Baseline 107.6 ± 26.9 vs 105.1 ± 20.9; 6-months 109.7 ± 26.3 vs 106.7 ± 22.1, p=0.59; 12-months 109.2 ± 27.6 vs 106.9 ± 22.5, p=0.54<br>BMI: Baseline 37.2 ± 8.3 vs 37.1 ± 7.4; 6-months 37.8 ± 8.1 vs 37.5 ± 7.7, p=0.57; 12-months 38.3 ± 8.7 vs 37.6 ± 7.2, p=0.52<br><br>Within-group difference<br>≥ 5% body weight loss: significant within-group effect at 6 months and 12 months for both group intervention and one-on-one intervention (all p<0.001)	✓	✓<br>(clinically signficant within group)
		**CVD**<br>Between-group difference<br>Systolic BP: Baseline 115.1 ± 12.8 vs 116.4 ± 15.1; 6-months 115.9 ± 13.4 vs 115.7 ± 12.1, p=0.33; 12-months 114.2 ± 13.3 vs 115.6 ± 13.1, p=0.98<br>Diastolic BP: Baseline 77.6 ± 8.4 vs 78.8 ± 10.8; 6-months 79.9 ± 9.9 vs 77.7 ± 9.8, p=0.03; 12-months 77.3 ± 10.6 vs 77.3 ± 10.6, p=0.98<br>Total cholesterol (mg/dL): Baseline 166.0 ± 41.8 vs 174.2 ± 45.9; 6-months 164.5 ± 40.4 vs 171.1 ± 41.3, p=0.50; 12-months 163.2 ± 39.8 vs 172.8 ± 54.1, p=0.38<br>LDL (mg/dL): Baseline 94.0 ± 32.3 vs 97.5 ± 35.6; 6-months 88.9 ± 25.8 vs 99.9 ± 36.1, p=0.06; 12-months 92.7 ± 27.4 vs 97.1 ± 46.6, p=0.47<br>HDL (mg/dL): Baseline 47.1 ± 18.1 vs 42.6 ± 13.5; 6-months 44.1 ± 12.2 vs 41.6 ± 9.3, p=0.48; 12-months 43.9 ± 11.2 vs 43.3 ± 11.8, p=0.34<br>HbA1c (%): Baseline 5.3 ± 0.8 vs 5.3 ± 1.0; 6-months 5.3 ± 0.7 vs 5.3 ± 0.6, p=0.96; 12-months 5.6 ± 1.4 vs 5.5 ± 0.7, p=0.83	✓<br>CVD risk, lipids	✓<br>(clinically significant within group)

		Within-group difference<br>Clinically significant reduction in CVD risk (defined by authors as achieving either 5% weight loss from baseline or >50m increase from baseline on 6-MWT): significant within-group effect at 6 months and 12 months for both group intervention and one-on-one intervention (all p<0.05)<br><br>**Physical activity/ Sedentary behaviour**<br>Between-group difference<br>6-MWT: Baseline 1398 ± 296 vs 1424 ± 250; 6-months 1386 ± 296 vs 1426 ± 248 , p=0.79; 12-months 1357 ± 271 vs 1319 ± 332, p=0.32<br>IPAQ total vigorous score (log of MET min): Baseline 3.2 ± 3.4 vs 2.4 ± 3.3; 6-months 4.3 ± 3.7 vs 3.7 ± 3.7, p=0.25; 12-months 3.4 ± 3.6 vs 2.6 ± 3.3, p=0.37<br><br>Within-group difference<br>≥ 50m increase in 6-MWT: significant within-group effect at 6 months and 12 months for both group intervention and one-on-one intervention (all p<0.001)	✓	✓<br>(clinically significant within group)
Baker, 2014[5]	8 or 12 weeks (4 weeks post treatment)	Mean ± SD pre vs post score [Standardised effect size (95% CI)]<br><br>**Dietary**<br>ARFS total score = 33.2 ± 10.5 vs 38.2 ± 8.1, p=0.001 [-0.97 (-1.48, -0.45)]<br>ARFS sub-scale – fruit = 5.1 ± 3.1 vs 6.6 ± 2.9, p=0.008 [-0.73 (-1.25, -0.22)]<br>ARFS sub-scale – vegetables 12.2 ± 4.0 vs 13.5 ± 3.5 [-0.64 (-1.15, -0.130]<br>Fruit and veg (serves/day) 4.2 ± 2.0 vs 5.0 ± 1.5 [-0.40 (-0.91, 0.12)]<br><br>**Mental health**<br>BDI = 4.5 ± 3.3 vs 3.7 ± 2.8, p=0.149 [0.37 (-0.15, 0.88)]<br>EUROHIS-QoL = 25.6 ± 5.6 vs 28.4 ± 6.6 [-0.65 (-1.16, -0.13)]<br>GAF = 57.1 ± 6.7 vs 62.7 ± 8.9, p=0.017 [-0.65 (-1.12, -0.17)]<br><br>**Physical activity/ Sedentary behaviour**<br>Screentime (min/day) = 298 ± 200 vs 163 ± 107, p=0.007 [0.76 (0.24, 1.27)]<br>Weekday sitting (min/day) = 555 ± 191 vs 412 ± 211, p=0.008 [0.73 (0.22, 1.24)]<br>IPAQ walking (min/week) = 252 ± 353 vs 356 ± 470, p=0.099 [-0.42 (-0.94, 0.09)]	✓<br><br>✓<br><br>✓	✓<br><br>✓<br>QoL, GAF<br><br>✓<br>Screentime, sitting
Baker, 2015[6]	15 weeks (mid-intervention) and 12 months	Mean change (95% CI) at 15wks; 12months<br><br>**Dietary**<br>*Intervention group*:<br>Fruit (serves/day) = -0.0 (-0.4, 0.4), p=0.994; 0.0 (-0.3, 0.4), p=0.771<br>Veg (serves/day) = -0.1 (-0.5, 0.3), p=0.417; -0.4 (-0.9, 0.1); p=0.021	✓	x

Fruit and veg (serves/day) = -0.1 (-0.8, 0.5), p=0.544; -0.4 (-1.1, 0.3); p=0.106		
Control group:		
Fruit (serves/day) = 0.1 (-0.3, 0.5), p=0.569; -0.1 (-0.4, 0.3), p=0.631		
Veg (serves/day) = 0.1 (-0.4, 0.5), p=0.638; 0.0 (-0.5, 0.6), p=0.908		
Fruit and veg (serves/day) = 0.2 (-0.5, 0.8), p=0.494; -0.1 (-0.8, 0.7), p=0.830		
**Mental health**	✓	✓
Intervention group:		
BPRS-24 = -2.3 (-5.0, 0.5), p=0.032; -4.6 (-8.8, -0.4), p=0.005		
BDI-II = −2.8 (−6.0, 0.3), p=0.018; −3.8 (−8.1, 0.4), p=0.018		
SF-12 Mental Component Scale = 1.1 (−1.6, 3.8), p=0.290; 1.6 (−2.4, 5.6), p=0.287		
SF-12 Physical Component Scale = 1.5 (−0.4, 3.5), p=0.042; 1.0 (−2.4, 4.4), p=0.420		
IWOQOL-Lite = −3.8 (−8.0, 0.4), p=0.018; −8.4 (−18.7, 2.0), p=0.035		
GAF = 5.3 (1.6, 9.1), p<0.001; 9.9 (4.5, 15.2), p<0.001		
Control group:		
BPRS-24 = 0.6 (-2.5, 3.7), p=0.632; -0.5 (-4.5, 3.5), p=0.758		
BDI-II = −3.0 (−7.0, 0.9), p=0.046; −3.6 (−6.8, −0.3), p=0.005		
SF-12 Mental Component Scale = 0.3 (−2.5, 3.0), p=0.801; 0.8 (−2.9, 4.4), p=0.571		
SF-12 Physical Component Scale = −0.1 (−1.8, 1.7), p=0.913; 1.4 (−1.5, 4.3), p=0.200		
IWOQOL-Lite = −0.6 (−5.6, 4.4), p=0.755; −2.4 (−8.4, 3.6), p=0.286		
GAF = 3.9 (0.6, 7.1), p=0.002; 9.7 (5.3, 14.0), p<0.001		
**CVD**	✓	✓
Intervention group:		
10yr CVD risk (ASSIGN score) = −2.8 (−4.8, −0.7), p=0.001; −1.6 (−3.2, −0.0), p=0.009		
Control group:		
10yr CVD risk (ASSIGN score) = −2.0 (−3.6, −0.4), p=0.002; −0.7 (−2.4, 1.0), p=0.276		
**Physical activity/ Sedentary behaviour**	✓	x
Intervention group:		
IPAQ walking time (min/week) = −10.1 (−159, 139), p=0.860; −38.9 (−179, 101), p=0.464		
IPAQ sitting total (min/week) = −77.0 (−601, 447), p=0.700; −210.6 (−784, 363), p=0.333		
Control group:		
Walking time (min/week) = 39.6 (−173, 252), p=0.624; 96.3 (−113, 306), p=0.226		
Sitting total (min/week) = −294.4 (−843, 254), p=0.160; −27.3 (−628, 574), p=0.904		
NS difference between groups (group effect p>0.05 for all variables)		

Baker, 2018[7]	15 weeks (mid-intervention), and 12, 18, 24, 30 and 36 months	Mean change (95% CI) at 15wks; 12mo; 18mo; 24mo; 30mo; 36mo<br><br>**Dietary**<br>*Intervention group*:<br>Overall diet score = −0.2 (−0.7, 0.4), p=0.380; −0.1 (−0.7, 0.5), p=0.571; −0.5 (–1.2, 0.3), p=0.105; 0.3 (−0.5, 1.0), p=0.345; 0.1 (−0.7, 0.9), p=0.836; −0.4 (−1.1, 0.3), p=0.109<br>*Control group*:<br>Overall diet score = −0.1 (−0.8, 0.5), p=0.645; −0.3 (−1.0, 0.3), p=0.190; −0.6 (−1.2, 0.1), p=0.021; −0.4 (−1.0, 0.2), p=0.100; −0.4 (−1.1, 0.3), p=0.108; 0.0 (−0.6, 0.6), p=1.000	x	x
		**Mental health**<br>*Intervention group*:<br>BPRS-24 = −2.3 (−5.0, 0.5), p=0.032; −5.0 (−9.1, −0.9), p=0.002; −2.0 (−6.5, 2.5), p=0.238; −3.0 (−6.7, 0.7), p=0.037; −2.5 (−6.4, 1.5), p=0.103; −4.9 (−8.4, −1.5), p<0.001<br>BDI-II = −2.9 (−6.0, 0.3), p=0.018; −3.9 (−8.1, 0.2), p=0.014; −3.2 (−7.0, 0.5), p=0.025; −3.8 (−7.5, −0.1), p=0.008; −4.3 (−7.6, −0.9), p=0.001; −6.7 (−9.8, −3.5), p<0.001<br>SF-12 Mental Component Scale = 1.4 (−2.2, 5.1), p=0.308; 2.3 (−2.6, 7.1), p=0.217; 1.6 (−3.1, 6.3), p=0.368; 3.3 (−0.9, 7.4), p=0.040; 1.5 (−3.1, 6.1), p=0.388; 4.2 (0.3, 8.1), p=0.006<br>SF-12 Physical Component Scale = 1.6 (−1.2, 4.4), p=0.136; 1.4 (−2.6, 5.4), p=0.356; −0.5 (−5.3, 4.3), p=0.786; 2.0 (−2.5, 6.5), p=0.238; −0.7 (−5.0, 3.7), p=0.681; 2.6 (−1.9, 7.1), p=0.129<br>GAF = 5.3 (1.6, 9.1), p<0.001; 7.5 (2.7, 12.2), p<0.001; 6.3 (1.7, 10.9), p<0.001; 6.9 (2.9, 10.9), p<0.001; 4.1 (−0.2, 8.5), p=0.015; 0.6 (−4.2, 5.4), p=0.728<br>*Control group*:<br>BPRS-24 = 0.6 (−2.6, 3.7), p=0.632; −0.5 (−4.5, 3.5), p=0.758; 1.0 (−2.5, 4.6), p=0.442; −0.7 (−4.9, 3.6), p=0.687; −2.4 (−6.9, 2.1), p=0.164; 0.4 (−4.1, 5.0), p=0.794<br>BDI-II = −3.0 (−7.0, 0.9), p=0.046; −3.5 (−6.8, −0.2), p=0.006; −2.0 (−5.7, 1.7), p=0.154; −4.0 (−7.4, −0.6), p=0.003; −4.0 (−8.2, 0.2), p=0.014; −2.8 (−6.1, 0.5), p=0.028<br>SF-12 Mental Component Scale = 0.7 (−3.2, 4.6), p=0.625; 1.9 (−1.9, 5.7), p=0.182; 0.5 (−4.2, 5.1), p=0.798; 2.9 (−1.7, 7.5), p=0.098; 4.6 (−0.3, 9.5), p=0.015; 3.5 (−1.2, 8.3), p=0.051<br>SF-12 Physical Component Scale = 0.2 (−2.6, 3.0), p=0.866; 0.6 (−3.5, 4.8), p=0.675; −0.9 (−3.9, 2.2), p=0.445; 3.0 (−0.6, 6.6), p=0.030; 2.9 (−0.0, 5.7), p=0.010; 3.7 (0.1, 7.3), p=0.009<br>GAF = 3.9 (0.6, 7.1), p=0.002; 7.8 (4.2, 11.3), p<0.001; 5.7 (1.9, 9.5), p<0.001; 4.3 (−0.6, 9.1), p=0.023; 3.9 (−1.6, 9.3), p=0.063; −1.1 (−5.7, 3.6), p=0.539	✓	✓
		**Anthro**<br>*Intervention group*:<br>Waist circumference (cm) = −0.6 (−2.4, 1.2), p=0.373; −1.5 (−4.7, 1.6), p=0.192; 0.4 (−2.6, 3.4), p=0.744; −0.3 (−3.4, 2.8), p=0.813; 0.1 (−3.0, 3.3), p=0.91; 0.7 (−2.9, 4.2), p=0.618<br>*Control group*:	✓	x

		Waist circumference (cm) = −0.4 (−2.6, 1.9), p=0.651; −0.8 (−3.8, 2.2), p=0.498; −0.7 (−3.8, 2.4), p=0.557; 1.1 (−2.1, 4.3), p=0.359; 0.7 (−3.1, 4.4), p=0.635; −0.8 (−4.6, 2.9), p=0.553		
		**CVD**<br>*Intervention group*:<br>10yr CVD risk (ASSIGN) = −2.0 (−3.6, −0.5), p<0.001; −1.9 (−3.4, −0.3), p=0.003; −2.2 (−3.9, −0.5), p<0.001; −1.9 (−3.5, −0.4), p=0.002; −1.6 (−3.2, 0.0), p=0.010; −1.4 (−2.8, −0.1), p= 0.007<br>*Control group*:<br>10yr CVD risk (ASSIGN) = −1.2 (−2.3, −0.2), p=0.002; −1.2 (−2.4, 0.1), p=0.014; −0.9 (−2.2, 0.4), p=0.070; −1.1 (−2.4, 0.2), p=0.029; −0.7 (−2.0, 0.7), p=0.180; −0.9 (−2.1, 0.4), p=0.070	✓	✓
		**Physical activity/ Sedentary behaviour**<br>*Intervention group*:<br>IPAQ walking time (min/week) = −10.1 (−159.3, 139.2), p=0.860; −41.1 (−179.3, 97.1), p=0.433; 15.5 (−90.6, 121.6), p=0.699; 75.5 (−21.6, 172.7), p=0.043; −33.3 (−176.5, 109.9), p=0.538; 13.7 (−161.4, 188.8), p=0.836<br>IPAQ sitting total (min/week) = −62.0 (−576.9, 452.9), p=0.752; −216.3 (−781.2, 348.7), p=0.313; 175.8 (−478.4, 830.1), p=0.477; 122.6 (−547.6, 792.8), p=0.627; 281.9 (−307.7, 871.5), p=0.209; −61.8 (−803.0, 679.4), p=0.826<br>*Control group*:<br>IPAQ walking time (min/week) = 39.61 (−173.2, 252.4), p=0.624; 100.5 (−111.7, 312.8), p=0.213; −60.8 (−236.3, 114.6), p=0.360; 72.1 (−95.4, 239.6), p=0.257; −23.2 (−160.8, 114.5), p=0.655; −48.8 (−222.8, 125.3), p=0.459<br>IPAQ sitting total (min/week) = −294.4 (−842.9, 254.2), p=0.160; −18.7 (−629.0, 591.7), p=0.936; 417.4 (−186.7, 1021.5), p=0.071; −16.1 (−645.2, 613.0), p=0.946; 405.6 (−353.8, 1165.0), p=0.160; 549.1 (−150.6, 1248.7), p=0.04<br><br>NS difference between groups (group effect p>0.05 for all variables)	✓	x
Lee, 2020[8]	12 and 28 weeks	Mean pre vs post vs follow-up scores (SD not reported)<br>Group, Time and Group x Time p-values		
		**Anthro**<br>*Intervention group*:<br>Body weight (kg) = 76.3 vs 75.5 vs 75.1<br>BMI = 26.6 vs 26.3 vs 26.2<br>Waist circumference (cm) = 98.8 vs 95.2 vs 93.5<br>*Control group*:<br>Body weight (kg) = 66.9 vs 68.2 vs 68.4	✓	✓<br>BMI, waist circumference

		BMI = 24.2 vs 24.7 vs 24.7<br>Waist circumference (cm) = 92.8 vs 92.4 vs 92.3<br><br>Body weight: Group p=0.039, Time p=0.796, Group x Time p=0.63<br>BMI: Group p=0.043, Time p=0.788, Group x Time p=0.013<br>Waist circumference: Group p=0.231, Time p=0.003, Group x Time p=0.024		
		**CVD**<br>*Intervention group*:<br>Total cholesterol (mg/dL) = 163.9 vs 166.3 vs 160.3<br>HDL (mg/dL) = 41.3 vs 41.7 vs 44.0<br>LDL (mg/dL) = 107.5 vs 114.7 vs 95.4<br>*Control group*:<br>Total cholesterol (mg/dL) = 164.3 vs 149.4 vs 167.6<br>HDL (mg/dL) = 38.6 vs 35.8 vs 38.3<br>LDL (mg/dL) = 107.8 vs 94.9 vs 106.3<br><br>Total cholesterol: Group p=0.851, Time p=0.475, Group x Time p=0.102<br>HDL: Group p=0.150, Time p=0.145, Group x Time p=0.325<br>LDL: Group p=0.809, Time p=0.598, Group x Time p=0.082	✓	✓<br>(within-group)
Looijmans, 2019[9]	6 months (mid-intervention) and 12 months	Intervention effect β (95% CI) at 6 and 12 months<br>Group difference, and time effect at 6 months and 12 months p-values		
		**Anthro**<br>BMI: 0.27 (-0.32, 0.85), p=0.38; 0.18 (-0.49, 0.86), p=0.60<br>Group p=0.08, Time 6mo p=0.49, 12mo p=0.31<br>Waist circumference (cm): -0.15 (-2.49, 2.19), p=0.90; -1.03 (-3.42, 1.35), p=0.39<br>Group p=0.45, Time 6mo p=0.86, 12mo p=0.08	✓<br>WC	x
		**CVD**<br>Metabolic syndrome z-score: -0.25 (-0.69, 0.18), p=0.24; -0.30 (-0.66, 0.05), p=0.09<br>Group p=0.63, Time 6mo p=0.48, 12mo p=0.51	✓	x
Nicol, 2022[10]	12 weeks	**Anthro**<br>Mean weight change for total sample: –0.7 ± 15.4 lbs (range –20.5 to 58 lbs).<br>Significant group x time effect on weight ($F_{1,23}$ =17.98, p<0.001); participants with lower symptom severity and high treatment engagement had significant decrease in weight ($F_{1,16}$ =22.54, p<0.001); participants with high symptom severity and low treatment engagement had	✓	✓

		non-significant increase in weight ($F_{1,7} = 4.33$, p=0.08). No significant time x treatment setting effect ($F_{1,23} = 2.22$, p = 0.15), or LOCES score effect ($F_{1,22} = 0.02$, p=0.90), on weight outcomes.		
Temmingh, 2013[11]	12, 24, 36 and 48 weeks	Mean ± SD at baseline vs week 12 vs week 24 vs week 36 vs week 48, or mean change over time		
		**Mental health**<br>General health rating change over time: 0.40 (0.33, 0.48); log scale: β = 0.0067409; 95% CI, 0.0042026 to 0.0092792; p<0.001	✓	✓
		**Anthro**<br>Weight (kg): 87.4 ± 19.8 vs 84.9 ± 18.0 vs 83.6 ± 17.2 vs 82.5 ± 17.0 vs 82.6 ± 17.1<br>Weight loss over time: -4.8 kg (95% CI, -5.67, -3.82); log scale: β = 0.0004705; 95% CI, -0.0005319, -0.0004091; p<0.001<br>Waist circumference (cm): 101.5 ± 17.0 vs 97.9 ± 14.6 vs 95.7 ± 13.0 vs 96.4 ± 15.1 vs 94.7 ± 14.7<br>Waist circumference change over time: -6.8 cm (95% CI, -8.49, -5.19); β = -0.1529126; 95% CI, -0.1717103, -0.1341149; p<0.001<br>BMI category (% participants) - 18-24 kg/m^2: 15.9 vs 19.0 vs 18.9 vs 18.9 vs 21.3 vs; 25-29 kg/m^2: 31.9 vs 35.6 vs 36.9 vs 37.7 vs 36.3; ≥30 kg/m^2: 52.2 vs 45.4 vs 44.2 vs 43.4 vs 42.4<br>BMI change over time: log scale: β = -0.0004599; 95% CI, -0.0005197, -0.0004001; p<0.001	✓	✓

6-MWT, 6-minute walk test; ARFS, Australian Recommended Food Score; ASSIGN, ASsessing cardiovascular risk using Scottish Intercollegiate Guidelines Network; BDI, Beck Depression Inventory; BMI, Body Mass Index; BP, blood pressure; BPRS-24, Brief Psychiatric Rating Scale; CI, confidence interval; CVD, cardiovascular disease; EUROHIS-QoL, World Health Organization 8-item index quality of life measure; HbA1c, glycated haemoglobin; HDL, high-density lipoproteins; IPAQ, International Physical Activity Questionnaire; IQR, interquartile range; IWOQOL-Lite, Impact of Weight on Quality of Life-Lite; LOCES, Loss of Control Over Eating Score; LDL, low-density lipoproteins; MET, metabolic equivalent of task; MSQ, Medical Symptoms Questionnaire; PHQ-9, Patient Health Questionnaire-9; QoL, quality of life; SF-12, 12-Item Short Form Health Survey; SF-36, 36-Item Short Form Health Survey.

[a]Tick indicates there was a positive improvement in the outcome assessed, and the change may or may not be statistically significant, compared with baseline at any stage of the intervention, that is, during the intervention, immediately post-intervention, or at follow-up; x indicates there was no improvement or a negative effect in the outcome assessed.
[b]Tick indicates outcomes assessed and reported a statistically significant change (p < 0.05) compared with either the control or with baseline at any stage of the intervention, that is, during the intervention, immediately post-intervention, or at follow-up; x indicates the outcome assessed, but a significant change not reported.

Table S4. Summary of outcomes

**Author, year**	**Follow-up timepoints from baseline**	**Outcome variables measured**	**Positive change in outcome** [a] (Change from baseline in pre-post studies, or change from baseline in the intervention group in RCTs)	**Statistically significant change in outcome** [b] (Change from baseline in pre-post studies, or between group differences in RCTs)
**Dietary outcomes**				
Baker, 2014 [5]	8 or 12 weeks (4 weeks post treatment)	F&V intake<br>Diet quality	✓<br>✓	✓<br>✓
Baker, 2015 [6]	15 weeks (mid-intervention) and 12 months	F&V intake	✓	x
Baker, 2018 [7]	15 weeks (mid-intervention), and 12, 18, 24, 30 and 36 months	Diet quality	x	x
**Mental health outcomes**				
Abbott, 2020 [1]	9-10 weeks	Depressive symptoms<br>Symptom burden<br>QoL	✓<br>✓<br>✓	✓<br>✓<br>✓
Baker, 2014 [5]	8 or 12 weeks (4 weeks post treatment)	Depression scores<br>Global functioning<br>QoL	✓<br>✓<br>✓	x<br>✓<br>✓
Baker, 2015 [5]	15 weeks (mid-intervention) and 12 months	Psychiatric symptoms<br>Global functioning<br>QoL	✓<br>✓<br>✓	✓<br>✓<br>x
Baker, 2018 [7]	15 weeks (mid-intervention), and 12, 18, 24, 30 and 36 months	Depression scale<br>Global functioning<br>QoL	✓<br>✓<br>✓	✓<br>✓<br>✓ 24 & 36 months
Temmingh, 2013 [11]	12, 24, 36 and 48 weeks	General health (self-rated scale)	✓	✓
**Anthropometric outcomes**				
Aschbrenner, 2016a [2]	6 months	Body weight<br>BMI	✓<br>✓	✓<br>✓

Aschbrenner, 2016b [3]	6 months	Body weight<br>BMI	Data not reported<br>Data not reported	x<br>x
Aschbrenner, 2022 [4]	6 and 12 months	≥5% body weight loss from baseline	✓	✓
Baker, 2018 [7]	15 weeks (mid-intervention), and 12, 18, 24, 30 and 36 months	Waist circumference	✓	x
Lee, 2020 [8]	12 weeks and 28 weeks	Waist circumference<br>BMI	✓<br>✓	✓<br>✓
Looijmans, 2019 [9]	6 months (mid-intervention) and 12 months	Waist circumference<br>BMI	✓<br>x	x<br>x
Nicol, 2022 [10]	12 weeks	Body weight	✓	✓
Temmingh, 2013 [11]	12, 24, 36 and 48 weeks	Body weight<br>Waist circumference<br>BMI	✓<br>✓<br>✓	✓<br>✓<br>✓
**CVD outcomes**				
Aschbrenner, 2022 [4]	6 and 12 months	CVD risk reduction<br>(5% or greater weight loss or ↑ >50m on 6MWT)<br>Lipids (triglycerides, total cholesterol, HDL, LDL)<br>HbA1c<br>BP	✓<br>✓<br>x<br>x	✓<br>x<br>x<br>x
Baker, 2015 [6]	15 weeks (mid-intervention) and 12 months	CVD risk (ASSIGN score)	✓	✓
Baker, 2018 [7]	15 weeks (mid-intervention), and 12, 18, 24, 30 and 36 months	10-year CVD risk	✓	✓
Lee, 2020 [8]	12 weeks and 28 weeks	Lipids (triglycerides, total cholesterol, HDL, LDL)	✓	✓
Looijmans, 2019 [9]	6 months (mid-intervention) and 12 months	Metabolic syndrome z-score	✓	x
**Physical activity/Sedentary behaviour outcomes**				

Aschbrenner, 2016a [2]	6 months	Cardiorespiratory fitness (≥ 50m increase in 6-MWT)	✓	x
Aschbrenner, 2016b [3]	6 months	Cardiorespiratory fitness (≥ 50m increase in 6-MWT)	Data not reported	x
Aschbrenner, 2022 [4]	6 and 12 months	Cardiorespiratory fitness (≥ 50m increase in 6-MWT) Vigorous activity score	✓<br>✓	✓<br>✓
Baker, 2014 [5]	8 or 12 weeks (4 weeks post treatment)	Screentime<br>Walking time (min/week)<br>Sitting time (min/week)	✓<br>✓<br>✓	✓<br>✓<br>✓
Baker, 2015 [6]	15 weeks (mid-intervention) and 12 months	Walking time (min/week)<br>Sitting time (min/week)	✓<br>✓	x<br>x
Baker, 2018 [7]	15 weeks (mid-intervention), and 12, 18, 24, 30 and 36 months	Walking time (min/week)<br>Sitting time (min/week)	✓ 18, 24, & 36 months<br>✓ 15wks, 12 & 36 months	x<br>x

Anthro, anthropometric; BMI, body mass index; BP, blood pressure; CV, cardiovascular; CVD, cardiovascular disease; F&V, fruit and vegetable; HDL, high density lipoprotein cholesterol; LDL, low density lipoprotein cholesterol; N/A, not assessed; QoL, quality of life.

[a]Tick indicates there was a positive improvement in the outcome assessed, and the change may or may not be statistically significant, compared with baseline at any stage of the intervention, that is, during the intervention, immediately post-intervention, or at follow-up; x indicates there was no improvement or a negative effect in the outcome assessed.

[b]Tick indicates outcomes assessed and reported a statistically significant change ($p < 0.05$) compared with either the control or with baseline at any stage of the intervention, that is, during the intervention, immediately post-intervention, or at follow-up; x indicates the outcome assessed, but a significant change not reported.

Refererences

1. Abbott RD, Sherwin K, Klopf H, Mattingly HJ, Brogan K. Efficacy of a Multimodal Online Lifestyle Intervention for Depressive Symptoms and Quality of Life in Individuals With a History of Major Depressive Disorder. Cureus. 2020;12(7):e9061. https://dx.doi.org/10.7759/cureus.9061.
2. Aschbrenner KA NJ, Shevenell M, Kinney E, Bartels SJ. A pilot study of a peer-group lifestyle intervention enhanced with mHealth technology and social media for adults with serious mental illness. J Nerv Ment Dis. 2016a;204(6):483-6. https://dx.doi.org/10.1097/NMD.0000000000000530.
3. Aschbrenner KA NJ, Shevenell M, Mueser KT, Bartels SJ. Feasibility of behavioral weight loss treatment enhanced with peer support and mobile health technology for individuals with serious mental illness. Psychiatr Q. 2016b;87(3):401-15. https://dx.doi.org/10.1007/s11126-015-9395-x.
4. Aschbrenner KA NJ, Gorin AA, Mueser KT, Browne J, Wolfe RS, Xie H, et al. Group Lifestyle Intervention With Mobile Health for Young Adults With Serious Mental Illness: A Randomized Controlled Trial. Psychiatr Serv. 2022;73(2):141-8. https://dx.doi.org/10.1176/appi.ps.202100047.
5. Baker AL, Turner A, Kelly PJ, Spring B, Callister R, Collins CE, et al. 'Better Health Choices' by telephone: A feasibility trial of improving diet and physical activity in people diagnosed with psychotic disorders. Psychiatry Res. 2014;220(1-2):63-70. https://dx.doi.org/10.1016/j.psychres.2014.06.035.
6. Baker AL, Richmond R, Kay-Lambkin FJ, Filia SL, Castle D, Williams JM, et al. Randomized controlled trial of a healthy lifestyle intervention among smokers with psychotic disorders. Nicotine Tob Res. 2015;17(8):946-54. https://dx.doi.org/10.1093/ntr/ntv039.
7. Baker AL, Richmond R, Kay-Lambkin FJ, Filia SL, Castle D, Williams JM, et al. Randomised controlled trial of a healthy lifestyle intervention among smokers with psychotic disorders: Outcomes to 36 months. Aust N Z J Psychiatry. 2018;52(3):239-52. 10.1177/0004867417714336.
8. Lee K, Choi HS, Han M. Effects of therapeutic lifestyle change mentoring on cardio-metabolic factors for schizophrenia. Arch Psychiatr Nurs. 2020;34(1):19-26. https://dx.doi.org/10.1016/j.apnu.2019.12.006.
9. Looijmans A, Jörg F, Bruggeman R, Schoevers RA, Corpeleijn E. Multimodal lifestyle intervention using a web-based tool to improve cardiometabolic health in patients with serious mental illness: results of a cluster randomized controlled trial (LION). BMC Psychiatry. 2019;19(1):339. https://dx.doi.org/10.1186/s12888-019-2310-5.
10. Nicol G, Jansen M, Haddad R, Ricchio A, Yingling MD, Schweiger JA, et al. Use of an Interactive Obesity Treatment Approach in Individuals With Severe Mental Illness: Feasibility, Acceptability, and Proposed Engagement Criteria. JMIR Form Res. 2022;6(12):e38496. https://dx.doi.org/10.2196/38496.
11. Temmingh H, Claassen A, van Zyl S, Carrara H, Dayakalashe NM, Myer L, et al. The evaluation of a telephonic wellness coaching intervention for weight reduction and wellness improvement in a community-based cohort of persons with serious mental illness. J Nerv Ment Dis. 2013;201(11):977-86. https://dx.doi.org/10.1097/NMD.0000000000000036.
